# On the Origin of Thermally Enhanced Upconversion Luminescence in Lanthanide-Doped Nanosized Fluoride Phosphors

**DOI:** 10.3390/ma18122700

**Published:** 2025-06-08

**Authors:** Shirun Yan

**Affiliations:** Department of Chemistry, Fudan University, Shanghai 200438, China; sryan@fudan.edu.cn

**Keywords:** thermal enhancement of upconversion luminescence, fluoride nanophosphors, validity assessment, experimental data quality

## Abstract

Thermally enhanced upconversion luminescence (UCL), also known as negative thermal quenching of UCL, denotes a continuous increase in the UCL emission intensity of a particular phosphor with a rising temperature. In recent years, the thermal enhancement of UCL has attracted extensive research attention, with numerous reports detailing this effect in phosphors characterized by varying particle sizes, architectures, and compositions. Several hypotheses have been formulated to explain the underlying mechanisms driving this thermal enhancement. This paper rigorously examines thermally enhanced UCL in fluoride nanoparticles by addressing two key questions: (1) Is the thermal enhancement of UCL an intrinsic feature of these nanoparticles? (2) Can the proposed mechanisms explaining this enhancement be unequivocally supported by the existing literature? Upon analyzing a compilation of experimental observations alongside the concurrent phenomena occurred during spectral measurements, it is postulated that thermally enhanced UCL intensity is likely a consequence of multiple extrinsic factors operating simultaneously at elevated temperatures, rather than being an intrinsic property of nanoparticles. These factors include moisture desorption, laser-induced local heating, and lattice thermal expansion. The size-dependent properties of nanoparticles, such as surface-to-volume ratio, thermal expansion coefficient, and quantum yield, are the fundamental reasons for the size-dependent thermal enhancement factor of UCL. Temperature-dependent emission spectral intensity is not a dependable indicator for assessing the thermal quenching properties of phosphors. This is because it is influenced not only by the phosphor’s quantum yield, but also by various extrinsic factors at high temperatures. The nonlinear nature of UCL further magnifies the impact of these extrinsic factors.

## 1. Introduction

Upconversion nanoparticles (UCNP) represent a class of luminescent nanomaterials that convert low-energy photons into high-energy luminescent emissions [1]. Their unique properties, such as large anti-Stokes shift, sharp emission spectra, long excited-state lifetime, and robust photostability, render UCNP as highly desirable for diverse applications such as bioimaging, theranostics, photovoltaics, anti-counterfeiting, and display technology [2,3,4,5]. Notably, lanthanide ions, characterized by their abundant ladder-like 4f energy levels and prolonged excited-state lifetimes, demonstrate effectiveness in achieving photon upconversion when embedded in hosts featuring low cut-off phonon energy [6]. This process involves the sequential absorption of low excitation energies, followed by the emission of high-energy photons. Specifically, the ion Yb^3+^, featuring an absorption band at approximately 980 nm, serves as a prototypical sensitizer ion. When paired with activator ions like Ho^3+^ or Er^3+^, it facilitates the production of green and red emissions. Alternatively, when combined with Tm^3+^, it results in violet emissions.

Upconversion mechanisms can be generally categorized into excited-state absorption (ESA), energy transfer upconversion (ETU), photon avalanche (PA), cooperative energy transfer (CET), and energy migration-mediated upconversion (EMU). Among these five mechanisms, ETU has been demonstrated to be the most efficient process [7,8]. The energy transfer from the sensitizer to activator always occurs in a phonon-assisted manner due to the mismatch in energy levels between sensitizers and various activators coupled with the parity-forbidden nature of f-f transitions.

In UCNP, numerous competing processes occur simultaneously, including radiative and nonradiative relaxation, as well as various energy transfers, such as phonon-assisted energy transfer from the sensitizer to the activator, energy transfer to high-energy vibrations on the surface or in the environment, cross-relaxation, and back-transfer. All of these processes play a vital role in determining the luminescence efficiency and relative intensities at different wavelengths. The luminescence efficiency of UCNP is determined by a delicate balance between these different relaxation and energy-transfer processes [8,9,10,11,12]. It is well known that the luminescence efficiency of UCNP is lower than that of their bulk counterparts. The key reason for this is a higher density of surface bound activators in UCNP due to the size effect, by which the excitation energy is transferred from the optically active lanthanide ions to defects, surface ligands, and surrounding solvent molecules with high-energy vibrational modes non-radiatively. The addition of an inert shell can effectively remove surface defects and isolate the optically active lanthanide ions from the surrounding high-energy vibrations, thereby reducing surface-related quenching. Consequently, the luminescence efficiency of the UCNP is improved [10,13,14,15].

Photoluminescence quantum yield (QY), defined as the number of emitted photons per number of absorbed photons, is the direct measure of the luminescence efficiency of UCNP. Given that upconversion luminescence (UCL) of UCNP generally involves nonlinear photophysical processes through the sequential absorption of excitation photons by the sensitizer lanthanide ion and energy transfer to the activator ions, the QY of UCNP is excitation power-dependent, and saturation effects occur upon increasing the excitation power density [1,16,17]. This behavior stands in stark contrast to downshifting luminescence (DSL),where QY remains unaffected by excitation power density, owing to the absence of nonlinear processes in the light conversion pathway [18,19].

It is generally recognized that the QY of a phosphor remains essentially constant over a considerable range of temperatures up to a certain point (the “thermal breakpoint”), beyond which the QY decreases quite rapidly as the temperature increases. This is because the QY is governed by the balance between the radiative and non-radiative decay rates from the upper emitting level of the phosphor. Often, the radiative decay rate remains temperature-independent, whereas the nonradiative rate commonly increases above a critical temperature. The phenomenon where the QY of a phosphor decreases with increasing temperature is termed thermal quenching (TQ). TQ has been naturally entangling with luminescence since its discovery. In conventional principles, there are barely any phosphors that could be immune from luminescence TQ [20].

Over the previous two decades, a considerable number of articles have been published reporting negative TQ (NTQ) phenomena in UCNP. NTQ refers to the continuous enhancement of the integrated emission spectral intensity of a specific phosphor as the temperature rises. Alternatively, this phenomenon has been termed anomalous TQ [21], inverse TQ [22], or thermally boosted (enhanced) UCL [23,24] in various publications. NTQ has garnered considerable attention in recent research, with numerous phosphors reported to exhibit this effect [21,22,25,26,27,28]. The thermal enhancement factor of UCL, *I*_max_(*T*)/*I*(*T*_0_), has been reported to range from several-fold [25,26] to as high as 1000-fold [29] and even 2000-fold [27]. Several explanations have been put forward to interpret these experimental observations. The recent advancements and challenges related to the NTQ of UCL have been comprehensively summarized in several recently published monograph reviews and perspective articles [30,31,32,33]. For the sake of brevity and clarity in subsequent discussions, only a brief survey is made of the typical results and explanations of this phenomenon reported or endorsed by more than one independent research group herein.

## 2. A Brief Survey of the Typical Results and Explanations of the Thermal Enhancement of UCL

An early work on this issue authored by Santos et al. was published in 1999 [34]. They reported a three-fold UCL enhancement in Er^3+^/Yb^3+^ co-doped Ga_2_S_3_-La_2_O_3_ chalcogenide glass in visible emission upon excitation at 1064 nm when heating the sample from 300 to 428 K. Several independent research groups have documented thermal enhancements of UCL above room temperature in various bulk materials, including Er^3+^/Yb^3+^ co-doped YAG ceramics [35], Yb^3+^/Nd^3+^ co-doped La_2_O_3_ microcrystals [36], Nd^3+^/Yb^3+^ co-doped oxyfluoride glass ceramics [37], Yb^3+^/Nd^3+^ co-doped LiYGeO_4_ [38], Yb^3+^/Nd^3+^ co-doped NaY(MoO_4_)_2_ [39], etc. Notably, an astonishing 1683-fold thermal enhancement of NIR emissions over the temperature range of 280–490 K was recorded in NaY(MoO_4_)_2_:Yb^3+^/Nd^3+^ [39]. These authors all ascribed the thermal enhancement of UCL to the temperature-dependent multiphonon-assisted anti-Stokes sideband excitation of a Yb^3+^ sensitizer.

In 2005, Suyver et al. published a study on the temperature-dependent UCL of β-NaYF_4_:18%Yb^3+^/2%Er^3+^ powder with a mean size of 1–3 μm across the temperature range of 10–200 K. There was shown to be an UCL enhancement phenomenon as the temperature increased from 10 to 100 K, keeping almost constant when the temperature further increased to 200 K [40]. Based on high-resolution excitation spectra, along with the Yb^3+^ and Er^3+^ energy level structures, the authors explained that the huge temperature dependence observed for all emission bands was due to efficient energy transfer from the second crystal field level of the ^2^F_5/2_|1> multiplet in Yb^3+^ to the lowest level of the ^4^I_11/2_ multiplet in Er^3+^ and efficient UCL on Er^3+^. Additionally, several studies reported thermal enhancements of UCL at cryogenic temperatures in β-NaYF_4_:18%Yb^3+^/2%Er^3+^ UCNP of approximately 25 and 45 nm in size, as well as in its bulk counterpart [41], NaYF_4_:10%Yb^3+^/0.72%Er^3+^ UCNP with dimensions of around 120 nm in length and 50 nm in width [42], and NaY_0.2_F_4_:Yb^3+^:Er^3+^:Gd^3+^_0.8_ UCNP with a TEM size of (46 ± 7) nm [43]. The main reason for this enhancement was explained as the preferable population of the slightly higher energy level of Yb^3+^ at high temperatures determined by the Maxwell–Boltzmann distribution, which is more energy-resonant with the Er^3+^ state.

Since 2014, Shao’s group has published a series of papers investigating the thermally enhanced UCL [21,44,45,46,47,48,49,50,51,52,53,54]. These studies have explored the impact of numerous factors associated with the UCNP, such as emitter types, particle sizes, architectures (whether core-only or core/shell, as well as the nature and thickness of the shell), morphologies (nanoparticles, nanowires, or nanorods), and the associated measurement conditions (measuring environments, excitation power density) on the thermal enhancement of UCL. The initial work by Shao’s group reported a size-dependent thermal enhancement of UCL for small-size β-NaYF_4_:20%Yb^3+^/2%Er^3+^ UCNP (<30 nm) in the temperature region higher than room temperature (300–450 K) both for core-only and core/shell UCNP. Notably, for 32 nm UCNP, the increase in the UCL intensity with temperature was almost negligible. When the particle size was smaller than 32 nm, the UCL enhancement became more significant with the reductions in particle size. The authors proposed that the phonon confinement effect, unique to small-sized particles, alters the phonon density of states, thereby influencing the Yb^3+^-to-Er^3+^ energy transfer rates and enhancing UCL. The phonon density of states (PDOS) in small UCNP are discrete and the low energy modes of PDOS are cutoff at low temperatures. As temperature increases, this phonon confinement effect is weakened, and thus more phonons become available to make up the energy mismatch between Yb^3+^ sensitizers and Er^3+^ activators, resulting in UCL enhancement with temperature in small-sized UCNP [21].

**Figure 1:**
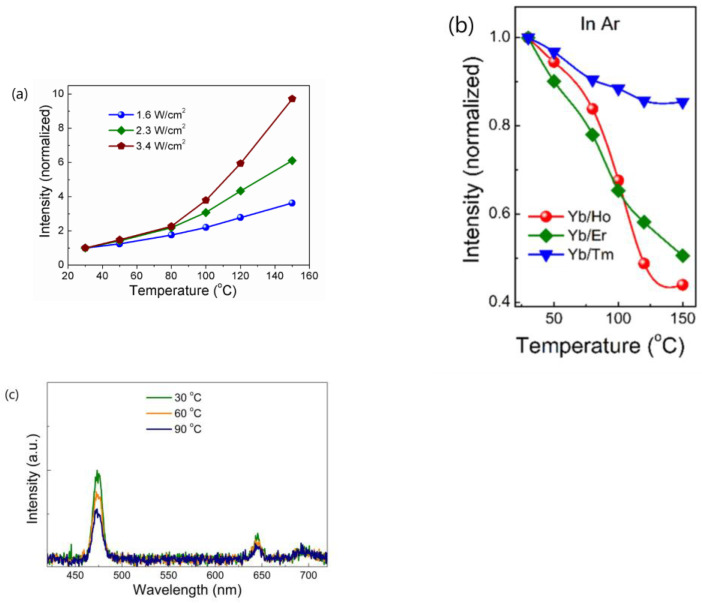
(**a**) Temperature-dependent UCL integrated intensities at various excitation power densities for NaGdF_4_:Yb/Tm small nanoparticles (~7 nm) synthesized using 10 mL of OA and 15 mL of ODE [44]. (**b**) UCL integrated intensities of 20%Yb/2%Ho, 20%Yb/2%Er and 20%Yb/2%Tm co-doped NaGdF_4_ small nanoparticles (~7 nm) as a function of temperature in Ar. Reproduced from Ref. [44] with permission. Copyright (2018) American Chemical Society. (**c**) Temperature-dependent UCL spectra of NaGdF_4_:Yb/Tm core-only UCNPs dispersed in the ODE solution. Reproduced from Ref. [51] with permission. Copyright (2017) The Royal Society of Chemistry.

Subsequently, the same authors revisited their rationale by analyzing the temperature-dependent emissions of NaGdF_4_:Yb^3+^/Ln^3+^ (Ln = Er, Tm, Ho) UCNP under different atmospheres and by dispersing the UCNP in 1-octadecene (ODE) solvent. They also examined the temperature-dependent downshifting luminescence (DSL) of Yb^3+^ at ~1050 nm. The results showed an anomalous enhancement of both UCL and DSL intensities coupled with an prolonged lifetime of Yb^3+^ DSL with increasing temperature for UCNP with larger surface-to-volume ratio and an active core/active shell. However, the enhancement factors for DSL of Yb/Ln co-doped small-NPs at 423 K were evidently lower than the corresponding UCL enhancement factors. The enhancement factor was found to be dependent on the excitation power, with a more significant enhancement observed under high-power excitation compared to low-power excitation, as illustrated in Figure 1a [44]. The thermal enhancement factors were also dependent on the emitter types, with the intensity increment of Tm^3+^ and Er^3+^ emissions at elevated temperatures being much lower than that of Ho^3+^ emissions for UCNP of similar sizes [44]. Moreover, thermal enhancement was observed only when the measurement was conducted in air or Ar/H_2_O atmospheres, and the thermal enhancement disappeared when the measurement was performed in an Ar atmosphere or when UCNP were dispersed in ODE, as shown in Figure 1b,c [44,51]. The authors discarded their prior interpretation of the phonon confinement effect and instead attributed the thermally induced luminescence enhancement to the thermal desorption of water molecules, which reduced quenching caused by –OH groups located on the surface [44,50,51]. In a recent publication, the same authors emphasized that the thermally enhanced UCL phenomenon was accompanied with additional temperature-dependent emission features, including reversibility, the increased intensity and prolonged decay time of the Yb^3+^ emission at elevated temperatures, and size/shell-layer/atmosphere dependencies [54]. The explanation that the desorption of surface-adsorbed water was responsible for the thermally enhanced UCL was also adopted by Meijerink’s group for Yb^3+^/Ln^3+^ (Ln^3+^ = Tm^3+^, Ho^3+^, Er^3+^) co-doped NaY(WO_4_)_2_ UCNP [28] and by several other research groups for Yb^3+^/Ln^3+^ co-doped fluoride UCNP [55,56,57].

In 2017, Wang’s group reported a reversible thermally enhanced UCL in β-NaGdF_4_:Yb^3+^/Ln^3+^ (Ln = Eu/Tb/Er/Tm) UCNP [25]. Specifically, the ^5^D_0_ → ^7^F_2_ emission intensity of 10 nm sized β-NaGdF_4_:20%Yb^3+^/10%Eu^3+^ exhibited a remarkable 16-fold enhancement as the temperature increased from 303 to 423 K. The enhancement was found to be particle-size-dependent, with smaller UCNP displaying a more significant effect. The critical size threshold for the disappearance of this abnormal thermo-enhanced UCL phenomenon was 50 nm. Moreover, the DSL intensity of Yb^3+^ at 1020 nm under 980 nm excitation showed the similar thermo-enhancement trend, while the DSL intensity of Eu^3+^ under 311 nm excitation exhibited normal TQ. Based on the temperature-dependent X-ray diffraction patterns, which demonstrate the main reflections gradually shifting to the lower angles as the temperature increases, the authors attributed this abnormal thermal enhancement of UCL to the alleviation of energy migration-induced surface quenching, stemming from the reduced Yb^3+^-mediated energy migration efficiency due to lattice thermal expansion in the UCNP [25]. In a subsequent work, the same authors investigated the temperature-dependent UCL of NaGdF_4_@NaGdF_4_:Yb^3+^/Tm^3+^ inert-core/active-shell UCNP (with a variety of inert-core sizes), where both the sensitizer and activator are located in the shell area near the nanoparticle’s surface, to substantiate the mechanism they proposed [58]. The results showed that the inert-core/active-shell UCNP exhibited a stronger luminescence thermal enhancement tendency compared to the homogeneously doped core-only UCNP of the same size. Intriguingly, the luminescence thermal enhancement behavior of the inert-core/active-shell UCNP appeared to be core-size-dependent, which could hardly be explained by either a surface phonon-assisted mechanism or a surface moisture-release mechanism. Instead, the authors proposed that the alleviation of the surface quenching induced by lattice thermal expansion was responsible for the luminescence thermal behavior of the inert-core/active-shell NaGdF_4_@NaGdF_4_:Yb^3+^/Tm^3+^ UCNP [58]. This explanation was also adopted by Chen et al. as the primary reason for the thermal enhancement of UCL in NaYbF_4_:Er@NaLuF_4_ and NaYbF_4_:Er@NaLuF_4_:Yb UCNP [59].

In 2018, Jin’s group reported an astonishing 2000-fold enhancement in the blue emission at 450 nm for 9.7 nm sized NaYF_4_:49%Yb^3+^/1%Tm^3+^ UCNP synthesized using the organometallic method as the temperature increased from 300 to 453 K [27]. This enhancement factor increased with the concentration of Yb^3+^ doping. The size-dependent spectral investigation showed that the thermal enhancement factor increased with the decreasing particle size of the UCNP. Notably, the thermal enhancement of the UCL could be observed in NaYF_4_:49%Yb^3+^/1%Tm^3+^ UCNP, even with a size as large as 57 nm. The authors proposed that the heat-favorable phonons present on the surface of lanthanide-doped UCNP facilitated the sensitizer-to-activator energy transfer [27]. One year later, Jin and colleagues reported that OA (oleic acid)-capped β-NaYF_4_:20%Yb^3+^/6%Nd^3+^ UCNP, both 25 nm and 10 nm in size, had stronger thermally enhanced NIR–NIR anti-Stokes emissions from 980 nm to 750 nm and 803 nm compared to the OA-free samples under the same measurement conditions. This further confirmed the essential role of the surface phonons in enhancing UCL [60]. In a 2021 collaboration between Jin’s and Yan’s groups, investigating into the temperature-dependent lifetime and emission intensity of *β*-NaYb_x%_Y_1−x%_F_4_ nanocrystals with mean sizes of 10–16 nm over the temperature range 303–453 K, the authors concluded that the optically silent Yb^3+^ ion in *β*-NaYb_x%_Y_1−x%_F_4_ nanocrystals could be activated by the vibrations of surface-coordinated water molecules through Yb–O at elevated temperatures. This led to enhanced emission intensities and prolonged lifetime values for all of the samples. In essence, vibrations of the oxygen moiety chelating Yb^3+^ ions generated by Yb^3+^ with surface-chelating OA ligands or surface-coordinated water functioned as surface phonons to facilitate efficient energy transfer from the Yb^3+^ sensitizer to the activators [61]. The explanation that enhancement of the luminescence of ultra-small UCNP was enabled by surface phonon-assisted energy transfer was endorsed by Liu’s group [62].

**Figure 2:**
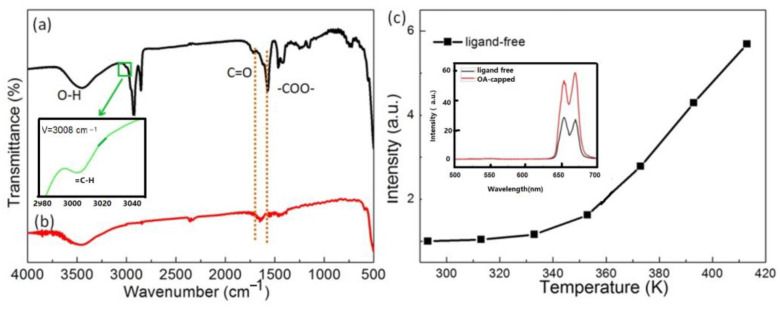
FTIR spectra of the OA-capped (**a**) and ligand-free (**b**) 20Yb/2Er: Na_3_ZrF_7_ NCs. (**c**) Dependence of the integral UC emission intensity on temperature for the ligand-free 20Yb/2Er: Na_3_ZrF_7_ NCs under 976 laser excitation. The inserts of (**c**) are the UC emission spectra of the OA-capped and ligand-free samples under the same characterization conditions. Reproduced from Ref. [22] with permission. Copyright (2018) The Royal Society of Chemistry.

In 2018, Xu and his colleagues reported a reversible thermally enhanced UCL of Ln^3+^ (Ln = Tm, Ho, Er) within a new-type fluoride system, specifically 20 nm sized Yb^3+^/Ln^3+^ co-doped Na_3_ZrF_7_ UCNP. The thermal enhancement of UCL was observed in both the ligand-free and inert-shell coated samples, as illustrated in Figure 2. Notably, the enhancement factor increased with a higher concentration of Yb^3+^ doping which, is consistent with the observations of Jin’s group [27]. Furthermore, the enhancement was dependent on the excitation power, exhibiting a more pronounced effect under high-power excitation compared to low-power conditions, in agreement with that observed by Shao’s group [44]. The authors suggested that the defect energy state originating from F^−^ vacancies in the Na_3_ZrF_7_ host, created by the aliovalent substitution of Zr^4+^ by Ln^3+^, could efficiently capture electrons from the excited states of the Yb^3+^ sensitizer. These electrons were then gradually released to the adjacent excited states of Er^3+^/Ho^3+^/Tm^3+^ activators as the temperature rose, leading to the significant enhancement of UCL [22]. Subsequently, the same research group reported that a more substantial UCL enhancement at elevated temperatures could be obtained in the NaGdF_4_@Ca/Yb/Er:NaGdF_4_ inert-core/active-shell system by introducing structural defects. They further noted that the UCL enhancement could be intensified by utilizing these defects as excitation energy reservoirs through hetero-valence substitution [63]. The notion that defects in the hosts contribute to the thermal enhancement of UCL was also supported by Rettori et al. [57] and Fu et al. [64].

In 2019, Zou et al. reported a reversible thermal enhancement of UCL in bulk materials featuring thermally induced lattice contraction [65]. As the temperature increased from 303 to 573 K, a remarkable 29-fold enhancement of Er^3+^ green UCL and 13-fold enhancement of overall UCL emission in orthorhombic Yb_2_W_3_O_12_ crystals doped with 6% Er^3+^ were observed. In contrast, Yb^3+^ DSL emission in the same crystals was appreciably quenched at high temperatures. The authors proposed that the thermal enhancement of UCL was primarily due to the enhanced energy harvesting by Er^3+^ activators at elevated temperatures. Specifically, lattice contraction at high temperatures narrowed the inter-ionic distance between Yb^3+^ and Er^3+^ ions, leading to the increase in energy transfer efficiency and, consequently, the enhancement of UCL. Notably, other research groups have also reported the thermal enhancement of UCL in hosts exhibiting thermally induced lattice contraction [66,67,68,69,70].

Based on the preceding discussion, it is evident that the thermal enhancement of UCL has been observed not only in nanosized materials, but also in bulk materials exhibiting either positive or negative thermal expansion coefficients. Furthermore, the temperature range within which this thermal enhancement is evident spans from cryogenic regions to the temperatures above room temperature. Various mechanisms have been proposed to explain this phenomenon. Notably, each of these proposed mechanisms for the thermal enhancement of UCL has garnered support from multiple research groups and appears to be backed by the experimental findings presented in their respective publications. The authors of these papers confidently assert that it is their proposed mechanism, rather than any other, that accounts for the experimentally observed thermal enhancement of UCL. There appears to be no single mechanism underlying thermally enhanced upconversion luminescence.

## 3. Potential Issues with Some Experimental Observations and Associated Explanations About Thermal Enhancement of UCL

A cursory review of the results and explanations outlined in these peer-reviewed articles reveals a plethora of conflicting experimental observations and mutually exclusive explanations for the identical luminescence thermal enhancement phenomenon observed in Ln^3+^-doped phosphors [31]. Upon examining a compilation of the relevant literature findings and mechanisms with respect to the coherence of experimental results and the compatibility of explanations for the same phenomenon, one may encounter considerable confusion regarding the credibility of both the results and the proposed mechanisms. It is difficult to understand how the temperature-dependent UCL intensity of a specific phosphor could diverge so significantly or how two contradictory mechanisms for the same phenomenon could be concurrently substantiated, as briefly discussed below.

**Figure 3:**
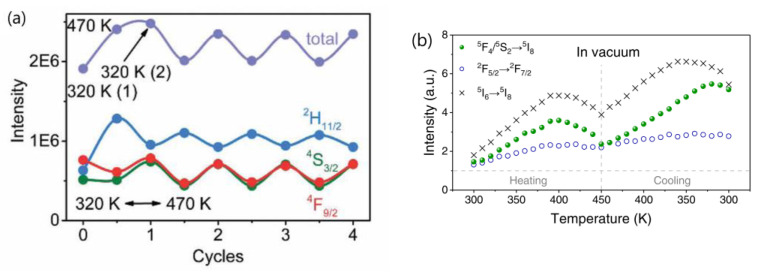
(**a**) Temperature-dependent luminescence of NaY(WO_4_)_2_:49Yb1Er NCs in dry nitrogen. Reproduced from Ref. [28] under terms of the CC BY 3.0 license. Copyright (2019) the Owner Societies. (**b**) Temperature-cycling experiment for thermally enhanced UCL in NaYF_4_:2%Ho,20%Yb@NaYF_4_:40%Yb NCs under 980 nm LD excitation in vacuum. Reproduced from Ref. [55] with permission. Copyright (2022) American Chemical Society.

1. The research conducted by Shao’s group indicated that the thermal enhancement of UCL above room temperature is a size-, morphology-, and architecture-dependent property of Yb^3+^/Ln^3+^ co-doped UCNP. This phenomenon was observed in core-only or active-core/active-shell structured UCNP, but not in their bulk and nanowire counterparts or UCNP with an active core/inert-shell architecture. Smaller UCNP exhibited a more significant enhancement factor. Shao’s group proposed that the thermal enhancement of UCL arises from the heat-induced recovery (or alleviation) of surface-related quenching, specifically the desorption of water molecules from the UCNP at elevated temperatures, which alleviates surface quenching and results in UCL enhancement [44,45,48,53,54]. Other researchers have also attributed thermally enhanced UCL to recovery of quenching due to the desorption of surface-adsorbed water [28,55,56,57]. However, if this mechanism holds true, one would expect the thermal enhancement factor to be associated with the desorption amount of water interacting with Ln^3+^ ions in the UCNP, which is dependent upon the specific surface area (surface-to-volume ratio) of the UCNP, but is independent of the experimental atmosphere. Contrary to this expectation, Shao’s group found that thermal enhancement of UCL was not observed when the ~10 nm sized core-only UCNP was dispersed in ODE or when the spectral measurement was conducted in an Ar atmosphere, as shown in Figure 1b,c. It is unclear why the desorption of water from the surface of the core-only UCNP did not occur upon heating in Ar or dispersing the UCNP in ODE. If the desorption of water could occur at elevated temperatures, it is perplexing why thermal enhancement was not observed in these conditions [44,51,52]. Measurements of the temperature-dependent spectra in controlled environments by Meijerink’s group and Zhang’s group showed that the thermal enhancements of UCL in 10 nm sized NaY(WO_4_)_2_:49%Yb^3+^/1%Er^3+^ and 42.7 nm sized NaYF_4_:2%Ho,20%Yb@NaYF_4_:40%Yb UCNP were still observed in the first heating cycle, even when the temperature-dependent spectral measurements were conducted in N_2_ or in a vacuum, respectively, as depicted in Figure 3 [28,55]. On the other hand, multiple independent researchers have demonstrated that the thermal enhancement of UCL can also be observed in active-core/inert-shell-structured UCNP [21,47,59,60,71]. The thermal enhancement of UCL in these UCNP cannot be attributed to the alleviation of surface quenching induced by the desorption of surface-adsorbed water, as the inert shell substantially suppresses surface-related quenching, as evidenced by the significantly higher QY values of core/shell UCNP compared to the core-only ones [10,13,14,15]. Furthermore, the alleviation of surface quenching induced by the desorption of surface adsorbed water can hardly explain why the thermal enhancement of UCL was not observed in nanowires and nanorods. Moreover, it is noteworthy that UCL thermal enhancement above room temperature was also observed in bulk materials like glass, ceramics, and microcrystals, even at temperatures of 800 K, substantially exceeding the threshold necessary for water desorption [34,35,36,37,72].

Many authors have proposed that the thermal enhancement of UCL in bulk materials exhibiting negative thermal expansion is predominantly due to the reinforcement of energy collection by Er^3+^ activators at elevated temperatures. As lattice contraction reduces the inter-ionic distance between Yb^3+^ and Er^3+^ ions at high temperatures, this leads to an improvement in energy transfer efficiency and, consequently, an enhancement of UCL [66,67,69,70,73,74,75,76,77]. Notably, a remarkable 1000-fold thermal enhancement of 611 nm emission in a bulk Y_2_W_3_O_12_:Nd phosphor was observed under 808 nm excitation, despite the fact that energy transfer is not required for UCL in this phosphor since Nd^3+^ is excited directly by the 808 nm radiation [78]. Conversely, some authors have suggested that the thermal enhancement of UCL in fluoride UCNP is due to the alleviation of the surface quenching induced by lattice thermal expansion [25,58,59]. However, it remains challenging to comprehend why both the negative and positive thermal expansion of the host lattice can be beneficial for energy transfer between the sensitizer and activator.

**Figure 4:**
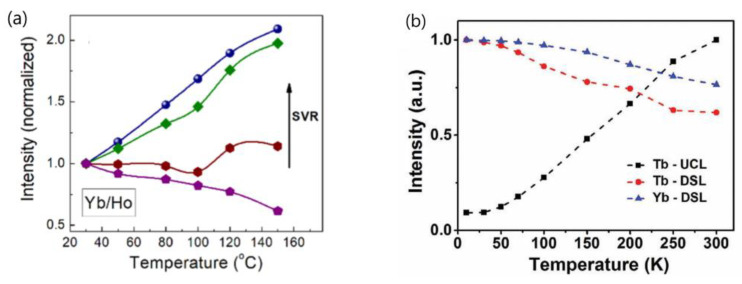
(**a**) Yb^3+^ DSL integrated intensities as a function of temperature for 20%Yb/2%Ho co-doped UCNP with various sizes. The intensities are normalized to that at 30 °C for each sample. Reproduced from Ref. [44] with permission. Copyright (2018) American Chemical Society. (**b**) Normalized integrated UCL intensity of Tb^3+^ and DSL intensities of Tb^3+^ and Yb^3+^ as a function of temperature for LiYbF_4_:30%Tb^3+^@LiYF_4_ core/shell UCNP upon NIR excitation at 980 nm. Reproduced under terms of the CC BY 3.0 license from Ref. [26]. Copyright (2017) The Royal Society of Chemistry.

**Figure 5:**
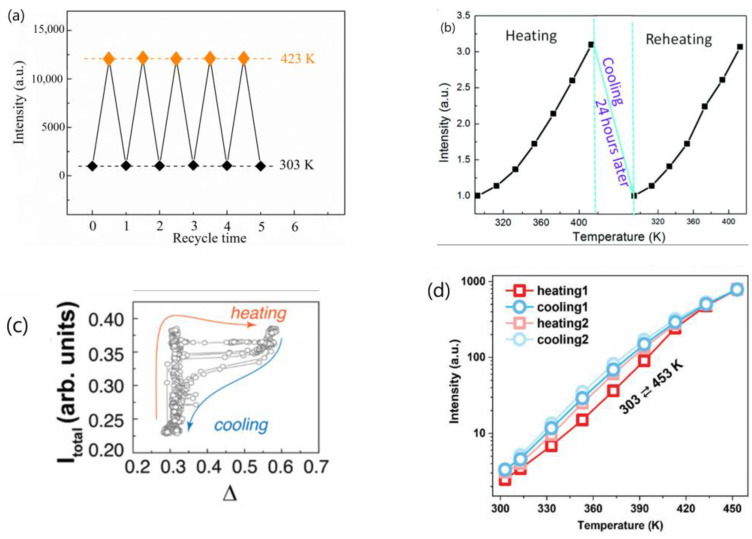
(**a**) Cycle measurements of abnormal thermo-enhanced UCL of NaGdF_4_:Yb^3+^/Eu^3+^ (20/10 mol %) lanthanide-doped nanoparticles with sizes of 10 nm. Reproduced from Ref. [25] with permission. Copyright (2017) The Royal Society of Chemistry. (**b**) A plot of the integral emission intensity of 20Yb/2Er:Na_3_ZrF_7_ NCs versus temperature measured during heating and reheating processes. Reproduced with permission from Ref. [22]. Copyright (2018) The Royal Society of Chemistry. (**c**) Evolution of the total integrated intensities (I_total_) in four thermal cycles for uncapped β-NaYF_4_ doped with 5 at% Yb^3+^ and 2 at% Er^3+^ nanoparticles; Δ is the thermometric parameter. Reproduced with permission from Ref. [57]. Copyright (2021) The Royal Society of Chemistry. (**d**) Dual-cycle heating–cooling test of the 10 nm NaYF_4_:60%Yb^3+^, 8%Nd^3+^ nanocrystals showing the release of the hysteresis effect during heating–cooling. Reproduced with permission from Ref. [60]. Copyright (2019) The Royal Society of Chemistry.

2. Wang’s group conducted a comprehensive investigation into the temperature-dependent emission intensity of both DSL of Yb^3+^ and UCL of Eu^3+^ and Tm^3+^ in NaGdF_4_:20%Yb^3+^/Eu^3+^ and NaGdF_4_@ NaGdF_4_:20%Yb^3+^/1%Tm^3+^ UCNP, respectively. Their experimental findings revealed that, under 980 nm excitation, both the DSL intensity of Yb^3+^ at 1020 nm and UCL of intensity of Eu^3+^ and Tm^3+^ increased as the temperature rose from 303 to 423 K [25,58]. Shao et al. and Li et al. also reported the thermally enhanced intensities of both the DSL of Yb^3+^ at ~1050 nm and UCL of Ho^3+^, Er^3+^ and Tm^3+^ in NaGdF_4_:Yb^3+^/Ln^3+^ (Ln = Er, Tm, Ho) UCNP, as exemplified in Figure 4a [44,50,51,53,56]. Similar results were also noted in Al_2_(WO_4_)_3_:Yb^3+^/Er^3+^ phosphors by Liao et al. [79]. Conversely, Chen and colleagues observed a different variation trend of DSL and UCL when heating LiYbF_4_:30%Tb^3+^@LiYF_4_ core/shell UCNP from 10 K to 300 K; the integrated UCL intensity of Tb^3+^ increased by approximately one order of magnitude, while the integrated DSL intensities of Tb^3+^ and Yb^3+^ decreased with rising temperatures, as illustrated in Figure 4b [26]. The variability in temperature dependencies of DSL and UCL reported by different authors poses a challenge for our understanding. Furthermore, it is well-established that after the absorption of a 980 nm photon, the Yb^3+^ is excited from the ^2^F_7/2_ to ^2^F_5/2_ level. The absorbed energy by Yb^3+^ ion can be depleted through two pathways: transfer to a neighboring Ln^3+^ (Ln = Ho, Er, Tm, Eu) ion, leading to UCL; or radiative decay accompanied with DSL emission at 1020 nm. In essence, the DSL of Yb^3+^ and UCL of Ln^3+^ are competing processes that both depopulate the intermediate excited state of Yb^3+^ (^2^F_5/2_). An efficient DSL may hinder the achievement of high UCL [44,80]. Given the conservation of energy, it is perplexing how the enhancement of both Yb^3+^ DSL and UCL intensities of Ln^3+^ (Eu^3+^, Ho^3+^, Er^3+^, Tm^3+^) could occur simultaneously in these phosphors under the condition that absorbed energy maintains constant.

3. Shao’s group conducted a comparative analysis of the thermal enhancement factors associated with UCL in β-NaYF_4_ UCNP doped with various ion-couples, namely Yb^3+^/Ln^3+^(Ln = Ho, Er, Tm). Their findings revealed that the Yb^3+^/Ho^3+^ co-doped phosphor exhibited the highest thermal enhancement factor, demonstrating a remarkable 12.6-fold enhancement compared to the 4-fold enhancement of Yb^3+^/Tm^3+^ co-doped variant within the temperature range of 303–423 K [44]. Intriguingly, a similar ranking of thermal enhancement factors among these ion-couples was also noted in Yb^3+^/Ln^3+^(Ln = Ho, Tm) co-doped β-NaGdF_4_ UCNP by the same research group [48,51]. However, Jin’s group conducted an investigation into the ion-pair-dependent thermal enhancement factors of UCL in β-NaYF_4_ 49%Yb^3+^/1%Ln^3+^ (Ln = Ho, Er, Tm) UCNP and reported contrasting results. They found that the Yb^3+^/Tm^3+^co-doped phosphor demonstrated the highest thermal enhancement factor, with a 16-fold enhancement, while Yb^3+^/Ho^3+^ and Yb^3+^/Er^3+^ co-doped phosphors exhibited only 7-fold and 2-fold enhancements, respectively [27]. The significant discrepancy in the reported ion-couple-dependent thermal enhancement factors of UCL for the same phosphor by different research groups poses a challenge in our understanding of the underlying physical mechanisms responsible for these variations.
4. Many authors have asserted that the thermal enhancement of UCL is reversible, as evidenced in their studies, as exemplified in Figure 5a,b [22,23,25,28,54,65,79,81]. However, a contrasting viewpoint has been put forward by multiple independent researchers who have reported that the thermal enhancement of UCL is non-reversible, as exemplified in Figure 5c,d [24,57,60,82,83,84]. Even for the same bulk phosphor, Yb_2_W_3_O_12_:6%Er^3+^, the reversibility of thermal enhancement of UCL and DSL were distinct [65,84]. It is difficult to understand why such contrasting phenomena could take place for the thermal enhancement of UCL.

## 4. Assessment of the Credibility of the Experimental Observations and Associated Explanations About the Thermal Enhancement of UCL in Lanthanide-Doped Fluoride Nanophosphors

The points raised above suggest that the understanding of the physics underpinning thermally enhanced UCL is still very limited, despite great effort being made. The literature presents conflicting viewpoints regarding both the conditions under which thermally enhanced UCL occurs and the mechanisms responsible for this phenomenon. Specifically, some researchers contend that thermally enhanced UCL is solely observed in UCNP with particle sizes below a certain threshold and diminishes when the particle sizes exceed this critical value or are in the form of nanowires and nanorods [25,28,44,55,58]. Conversely, other studies report instances of thermally enhanced UCL in bulk materials [34,35,36,37,38,39]. Notably, none of the mechanisms proposed in the literature have garnered universal acceptance as a definitive explanation for all experimental observations of thermally enhanced UCL.

In light of the numerous diverging and conflicting experimental results reported about the thermal enhancement of UCL, often without acknowledging measurement uncertainty, and given the challenges associated with accurately measuring spectral intensity at different temperatures, which can introduce substantial errors [85,86,87,88,89], there is a pressing need to reassess the validity of the experimental findings and their associated mechanisms for thermally enhanced UCL presented in the current literature. We hope to provoke critical thinking about experimental design strategies by removing some often-overlooked pitfalls in quantitative temperature-dependent fluorescence intensity measurements.

To streamline and focus our discussion, we will concentrate specifically on the thermal enhancement of UCL in fluoride-based UCNP (especially, NaLnF_4_, where Ln = Y, Gd, Lu). These phosphors have been extensively researched by various groups, resulting in a wealth of data available in the literature.

### 4.1. Is the Thermal Enhancement of UCL an Intrinsic Property of Lanthanide-Doped Fluoride-Based UCNP?

It is generally accepted that an intrinsic property of materials, including the size-dependent property of nanomaterials, should be well-determined with consistent replicability. Replicability and predictability are two pivotal attributes of intrinsic properties. The size-dependent melting point of nanomaterials has been a subject of extensive research for a century [90], with Pawlow first predicting this phenomenon in 1909 [91] and Takagi experimentally validating it in 1954 [92]. It has been well established that metal nanoparticles typically exhibit a lower melting temperature compared to their bulk counterparts, attributed to the surface effect and quantum size effect. Furthermore, the melting temperature of nanoparticles demonstrates a nearly linear relationship with the inverse of their particle diameter [93,94].

**Figure 6:**
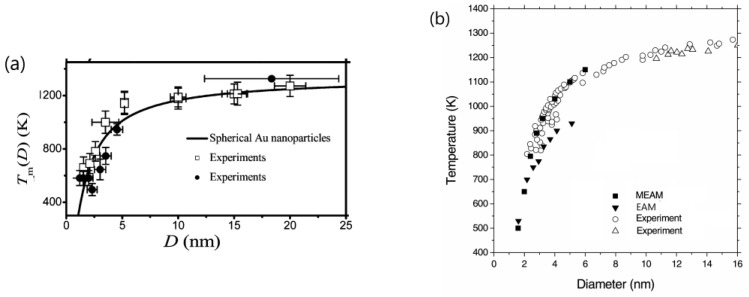
(**a**) Comparisons of T_m_(D) functions for spherical Au nanoparticles between the predictions and available experimental data: □ Ref. [95], • Ref. [96]. Adapted with permission from Ref. [105]. Copyright (2009) American Chemical Society. (**b**) Melting temperature as a function of particle diameter. ▼ EAM [103], ■ MEAM [104] refer to the embedded atom method and the modified embedded atom method, respectively. ο Ref. [97], △ Ref. [98]. Adapted with permission from Ref. [104]. Copyright (2002) Elsevier Science B.V.

Extensive studies have been conducted on the melting temperatures of gold nanoparticles using various experimental techniques, such as differential thermal analysis (DTA) coupled with thermal gravimetric analysis (TGA) [95], field-emission current density [96], diffraction of high-energy electrons [97], and electron microscopy [98,99,100], along with theoretical simulations [101,102,103,104,105,106,107]. However, due to variations in measurement methodologies and the different forms of gold nanoparticles (free-standing [100], silica-encapsulated [95], or supported [97,108]), the reported melting temperatures of gold nanoparticles for a specific particle size vary among authors. Despite these discrepancies, the experimental observations on the size-dependent melting temperatures of gold nanoparticles reported by different researchers align closely with theoretical predictions, as illustrated in Figure 6. Analogously, if the thermal enhancement of UCL is an intrinsic property of fluoride UCNP, the temperature dependence of UCL intensity of the given UCNP should be both predictable and replicable.

Table 1 presents a compilation of the experimental data regarding the temperature-dependent UCL intensity of fluoride UCNP as reported in the literature. For ease of comparison, samples sharing the same host composition and emitter type are listed together. The data in Table 1 are organized as follows: the UCL intensity of interest (*I*_x_), the thermal enhancement factor (calculated as *I*_max_(T)/*I*(T_0_)), the temperature range within which thermal enhancement has occurred (Δ*T*), the enhancement rate per unit temperature interval (Δ*I*/Δ*T*), the power or power density and wavelength of the incident excitation laser, and the size and architecture of the UCNP.

**Figure 7:**
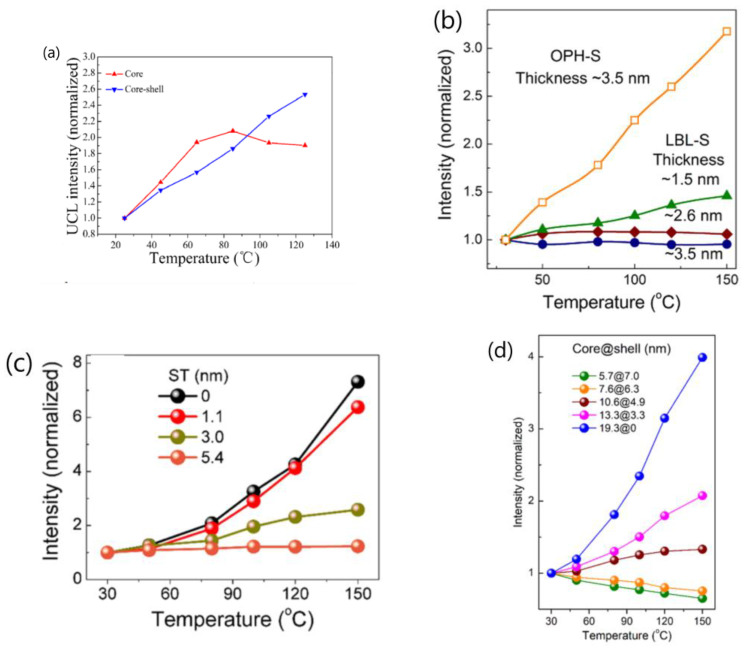
(**a**) Integrated UCL intensities of core-only and core–shell UCNPs as a function of temperature. Intensities were calculated by integrating the spectral intensity of the UCL spectra over a wavelength range of 510–680 nm and normalized to that of 25 °C for each sample. Reproduced from Ref. [21] with permission. Copyright (2014) American Chemical Society. (**b**) Temperature-dependent UCL intensities of NaGdF_4_:20%Yb/2%Tm@NaGdF_4_ core/shell UCNC synthesized via the successive layer-by-layer (LBL) or one-pot heating-up (OPH) method. Reproduced from Ref. [44] with permission. Copyright (2018) American Chemical Society. (**c**) Temperature-dependent UCL intensities of core-only and core/shell NCs with various inert-shell thicknesses (power density: 1.6 W·cm^−2^). Note that the intensity is normalized to that of 30 °C. Reproduced from Ref. [47] with permission. Copyright (2019) American Chemical Society. (**d**) Integrated UCL intensities of serial NaGdF_4_:Yb^3+^/Er^3+^@NaGdF_4_ nanocrystals upon 980 nm excitation at elevated temperatures from 30 to 150 °C. Reproduced from Ref. [49] with permission. Copyright (2022) American Chemical Society.

**Figure 8:**
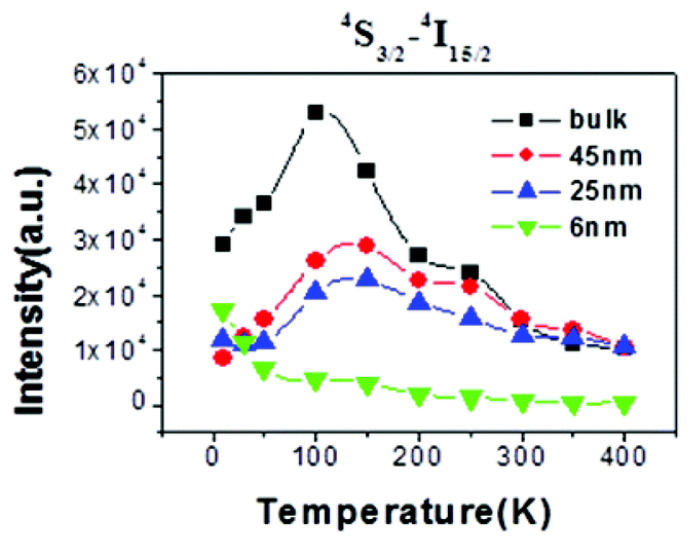
Difference in upconversion green emission intensity of NaYF_4_:Yb^3+^, Er^3+^ nanoparticles, and bulk when taken as dry powder and excited under 980 nm LD excitation with the same power density (2.9 W mm^−2^) at verified temperatures from 10 K to 400 K in vacuum. Reproduced with permission from Ref. [41]. Copyright (2014) The Royal Society of Chemistry.

One can receive following information from Table 1:

1. The majority of the researchers examined the temperature-dependent UCL spectra of solid powder samples directly [27,44,55,58,59,114]. Alternatively, some authors investigated the temperature-dependent UCL spectra of the UCNP colloidal solutions in organic solvents [42,51,81,112]. Notably, a significant thermal enhancement of UCL above room temperature was exclusively observed when the measurement was conducted using solid powder UCNP. Conversely, no any thermal enhancement was observed when the measurement was conducted on the colloidal solutions of both core-only and core/shell UCNP, and the luminescence intensities decreased at a higher temperature with the core-only UCNP decreasing more strongly [81,112].
2. The wavelength and power density of the excitation light chosen for temperature-dependent UCL spectral measurement varied among different researchers. Notably, some authors did not specify the power or power density of excitation light [24,48,50,63,109,114,115].
3. The temperature range where the thermal enhancement of UCL was observed varied considerably between papers by different authors. Some authors reported that the thermal enhancement of UCL was observed only at cryogenic temperatures, with thermal quenching initiating already at low temperatures [41,42]. However, other authors reported that the thermal enhancement of UCL was observed in the range from room temperature to 450 K [27,55,56], and even to 473 K [59].
4. The reported critical size of UCNP, beyond which UCL intensity changes from thermal enhancement to thermal quenching, varied significantly among different authors or different publications of the same research group. Shao and colleagues observed thermal enhancement exclusively in small-sized NaYF_4_:Yb^3+^/Er^3+^ UCNP (<30 nm), noting that the increase in UCL intensity with temperature for 32 nm UCNP was almost negligible [21]. In another paper authored by Shao and colleagues, strong thermal enhancement of UCL was still observed in NaYF_4_:20%Yb^3+^/2%Ho^3+^@NaYF_4_: 20%Yb^3+^ with a size of ~37.8 nm (core-size ~32.6 nm) [54]. Wang and co-workers reported that the critical size of β-NaGdF_4_:Yb^3+^/Ln^3+^ (Ln = Eu/Tb/Er/Tm) UCNP at which this abnormal thermo-enhanced UCL phenomenon ceased was 50 nm [25]. Conversely, Jin and co-workers found that the thermal enhancement of the UCL could occur even in NaYF_4_:49%Yb^3+^/1%Tm^3+^ UCNP with sizes of 57 nm [27]. On the other hand, Ji and colleagues reported that the thermal enhancement of UCL was observed in OA-removed NaYbF_4_:0.5%Tm^3+^ UCNP with sizes as large as 152 nm [118].
5. The architecture of UCNP exhibiting thermal enhancement of UCL was also considerable different in the reports. Shao and colleagues observed thermal enhancement only in core-only and active-core/active-shell UCNP, while thermal quenching of UCL was observed in UCNP with active-core/inert-shell architectures [44,46,51,53]. However, Jin et al. and Rettori et al. reported that thermal enhancement of UCL was also observed in the nanocrystals passivated with an inert shell [22,60,71].
6. The critical shell thickness in active-core/inert-shell-architectured UCNP, beyond which UCL intensity switches from thermal enhancement to thermal quenching, has been reported to span a wide range by the same research group in various publications. In 2014, Shao and co-workers observed UCL enhancement in NaYF_4_:Yb^3+^,Er^3+^ @NaYF_4_ core/shell UCNP with a core diameter of ~24 nm and a shell thickness of 2 nm, as illustrated in Figure 7a [21]. However, in subsequent reports in 2017 and 2018, they found no thermal enhancement in NaGdF_4_:Yb/Tm@NaGdF_4_ core/shell UCNP with a core diameter of ~8 nm and a shell thickness of 3.5 nm [51] or in NaGdF_4_:Yb^3+^/Er^3+^@NaGdF_4_ core/shell UCNP with a core diameter of ~7 nm and a shell thickness of 3.5 nm [44], as depicted in Figure 7b. In 2019, Shao and colleagues reported a critical “shell thickness (ST)” for thermal enhancement of UCL in NaGdF_4_:20%Yb^3+^/2%Er^3+^@NaGdF_4_ core/shell UCNP of 5.4 nm [47], as illustrated in Figure 7c, which was 1.5 times larger than the value reported in 2017 and 2018. More recently, in 2022, Shao’s group reported a “critical shell thickness” for the thermal enhancement of UCL in NaGdF_4_:20%Yb^3+^/2%Er^3+^@NaGdF_4_ of 4.9 nm, as shown in Figure 7d [49]. This value was evidently different from those reported previously.

7. The variation trends of UCL intensity with temperature for fluoride UCNP with a similar composition, particle size, and architecture, as reported by various research groups, exhibit notable discrepancies. Specifically, some researchers have documented that the UCL intensity of core-only NaYF_4_:Yb^3+^/Er^3+^ UCNP with a particle size of 30 nm [21,25] increases monotonously as the temperature rises above room temperature, as illustrated in Figure 7a. Conversely, other researchers have observed a steady decline in UCL intensity for NaYF_4_:Yb^3+^/Er^3+^ UCNP with particle sizes of 30–40 nm decreased steadily with increasing temperature [82,111,112]. In some cases, thermal quenching is evident even from cryogenic temperatures [41,109], as exemplified in Figure 8. Distinct from these two monotonic patterns, Tong et al. reported that the UCL intensity of α-NaYF_4_:Yb^3+^/Er^3+^UCNP with sizes of 75 nm decreased with an increase in the temperature from 303 to 483 K, but subsequently increased with further temperature elevation from 483 to 573 K [83].

**Figure 9:**
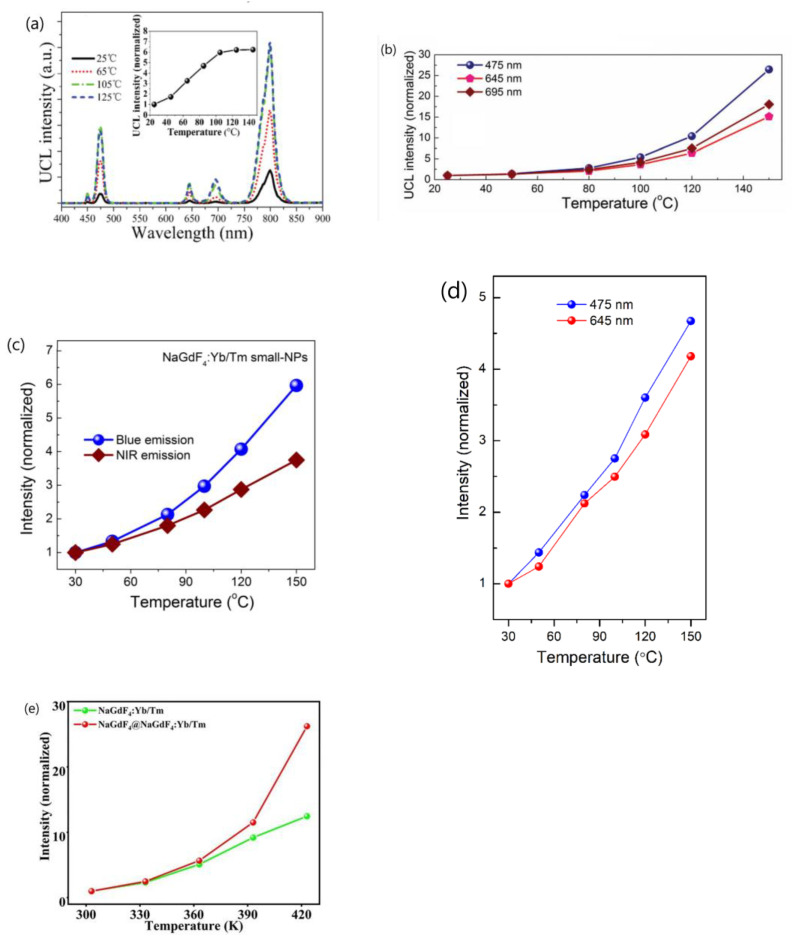
(**a**) Temperature-dependent UCL spectra of NaGdF_4_:20%Yb^3+^/1% Tm^3+^ nanoparticles (The corresponding integrated UCL intensities as a function of temperature are shown in the insets. Integrated intensities at various temperatures were normalized to that at 25 °C for each sample). Reproduced from Ref. [48] with permission, Copyright (2015) WILEY-VCH Verlag GmbH & Co. (**b**) UCL intensities of NaGdF_4_:20%Yb/1%Tm UCNPs as a function of temperature. Intensities are normalized to that at 25 °C for each emission band (power density: 4.7 W·cm^−2^). Reproduced with permission from Ref. [51]. Copyright (2017) The Royal Society of Chemistry. (**c**). Integral intensities of various UCL bands as a function of temperature for NaGdF_4_:Yb/Tm small nanoparticles. The intensities are normalized to that at 30 °C for each band. Reproduced from Ref. [44] with permission. Copyright (2018) American Chemical Society. (**d**) UCL spectra and integrated intensities of NaGdF_4_:20%Yb/1%Tm core nanocrystals. Reproduced with permission from Ref. [50]. Copyright (2023) The Royal Society of Chemistry. (**e**) Temperature-dependent integrated UCL intensities for NaGdF_4_:20%Yb/1%Tm (~12 nm) and NaGdF_4_@NaGdF_4_:20%Yb/1%Tm (~9@3 nm) UCNPs at elevated temperature from 30 °C to 150 °C in air. Reproduced with permission from Ref. [58]. Copyright (2021) The Royal Society of Chemistry.

The thermal enhancement factor and the rate of thermal enhancement per unit temperature interval of UCL intensity in specific UCNP, as reported by different researchers or in different publications from the same research group, exhibited significant variability. Taking Yb^3+^/Tm^3+^ co-doped fluoride UCNP as an illustrative example, Jin and co-workers reported [27] that, for 9.7 nm core-only β-NaYF_4_:49%Yb^3+^/1%Tm^3+^ UCNP, the blue emissions at 450 nm and 475 nm, corresponding to the Tm^3+^ transitions ^1^D_2_ → ^3^F_4_ and ^1^G_4_ → ^3^H_6_, respectively, were enhanced by factors of 2000 and 300 as the temperature increased from 303 to 453 K. In contrast, Shao and colleagues reported varying degrees of UCL enhancement in their studies. In a 2015 paper, they noted that the overall UCL intensity of ~8.4 nm core-only NaGdF_4_:20%Yb^3+^/1%Tm^3+^ UCNP increased by 6.2 times as the temperature increased from 298 to 498 K, as illustrated in Figure 9a [48]. In a 2017 publication, they observed 25-fold increase in the emission intensity of the 475 nm band for 9.5 nm core-only NaGdF_4_:20%Yb^3+^/1%Tm^3+^ UCNP over the temperature range of 298–423 K [51], as shown in Figure 9b. Furthermore, in a 2018 study, they reported a 6-fold increase in the intensity of the 475 nm blue emission for 7.0 nm core-only NaGdF_4_:20%Yb^3+^/2%Tm^3+^ small-NPs when the temperature was increased from 303 to 423 K, as illustrated in Figure 9c [44]. Most recently, in a 2023 paper, they found that, upon 975 nm excitation, the emission intensity of the 475 nm band of ~8.5 nm core-only NaGdF_4_:20%Yb^3+^/1%Tm^3+^ UCNP increased by 4.5-fold as the temperature rose from 303 to 423 K, as shown in Figure 9d [50].

Additionally, Wang et al. reported an 11-fold increase in the integrated UCL intensities of 12 nm core-only NaGdF_4_:20%Yb^3+^/1%Tm^3+^ UCNP as the temperature increased from 303 to 423 K, as shown in Figure 9e [58]. Qiu and colleagues observed a rapid increase in UCL intensity at 475 nm by over 24.2 times from the Tm^3+ 1^G_4_ → ^3^H_6_ transition when heating the as-prepared core-only (11.6 ± 1.6) nm NaGdF_4_:20%Yb^3+^/0.5%Tm^3+^ UCNP from room temperature to 463 K [24]. Li and co-workers reported a 17-fold thermal enhancement in the UCL integrated intensity of ~12.5 nm core-only NaGdF_4_:39%Yb^3+^/1%Tm^3+^ UCNP from 303 to 453 K [56].

The enhancement factors for the 475 nm UCL intensity in Yb^3+^/Tm^3+^ co-doped fluoride UCNP within a similar particle size range varied widely from several-fold to 300-fold.

Given these inconsistent experimental results in terms of either the critical size threshold, architecture of UCNP which exhibit thermal enhancement of UCL, or the thermal enhancement factor and the rate of thermal enhancement per unit temperature interval of UCL intensity in a specific UCNP, it seems challenging to confidently conclude that thermally enhanced UCL is an intrinsic and predictable or replicable property of these UCNP.

### 4.2. Can the Proposed Mechanisms Explaining the Enhancement Be Unequivocally Supported by the Existing Literature?

As detailed in the preceding section, multiple explanations have been proposed to elucidate the underlying mechanism behind the experimentally observed thermal enhancement of UCL intensity in fluoride UCNP. Several of these explanations, which have garnered support from more than one independent research group, are outlined below:

i. Preferable population of higher energy levels: At higher temperatures, there is a preferential population of the slightly higher energy level of Yb^3+^, which is more energy-resonant with the Er^3+^ state, thereby facilitating energy transfer from Yb^3+^ to Er^3+^ [26,40,41].
ii. Suppression of energy migration-induced surface quenching: Lattice thermal expansion of UCNP can suppress energy-migration-induced surface quenching [25,58,59].
iii. Suppression of the OH vibration-induced de-excitation: The desorption of moisture at elevated temperatures can suppress OH vibration-induced de-excitation of the Yb^3+ 2^F_5/2_ state [44,50,51,55,56,57].
iv. Surface phonons enhanced energy transfer: Surface phonons, generated by chelating ligands between Yb^3+^ and carboxylic moieties, favor the sensitizer-to-activator energy transfer [27,60,61,62].
v. Energy transfer from the defects: Energy transfer from defects can also contribute to the observed thermal enhancement [22,57,63,64].

Each of these explanations appears plausible within the context of their respective published papers. However, these explanations for the same experimental observation of thermal enhancement seem incompatible. An authentic and universal mechanism underpinning the thermal enhancement of UCL should be substantiated by the complex experimental observations reported in the literature. Can any of the above explanations be unequivocally supported by the reported results?

(i) The preferable population of the slightly high-energy multiplet of Yb^3+^ (^2^F_5/2_) at high temperatures, resulting in a more efficient Yb^3+^-to-Er^3+^ energy transfer [26,40,41].

This mechanism considers that the ^2^F_5/2_ energy level of Yb^3+^ is made of several multiplets, with the high-lying multiplet being more energy-resonant with the Er^3+^ state. When the incident photon can only afford the population of the lowest-lying multiplet ^2^F_5/2_∣0>, further populations of the high-lying multiplet ^2^F_5/2_∣1> can be realized via the absorption of multiple phonons by low-lying multiplet electrons, ultimately enhancing energy transfer from Yb^3+^ to Er^3+^ [26,40,41].

It is well-known that the energy levels of 4f electrons are largely shielded from environmental influences. As a result, the optical transitions on dopant ions are localized and remain unaffected by quantum size effects. Consequently, the multiplets of the ^2^F_5/2_ energy level of Yb^3+^ in nanosized materials are roughly the same as those in bulk materials. The phonon-assisted energy transfer alone fails to provide a satisfactory explanation for the size-dependent thermal enhancement of UCL, particularly the thermal enhancement of UCL observed across different temperature regions.

(ii) Suppression of energy migration to surface quenchers by lattice thermal expansion [25,58,59].

The rationale behind this mechanism is as follows: The phenomenon of size-dependent abnormal luminescence thermal enhancement in UCNP echoes other size-dependent properties, with the most well-known being size-dependent luminescence quantum yield (QY). It has been established that the QY of UCNP decreases with decreasing size due to the surface quenching of excitation energy. Given that UCNP doping strategies often involve introducing a high concentration of sensitizer (Yb^3+^) to provide a large absorption cross-section in the NIR region, the surface-quenching effect can be significantly amplified by the sensitizer-mediated “long-range” energy migration process. This energy migration process is highly sensitive to minute changes in the working distance. The lattice thermal expansion can exert a substantial influence on the efficiency of energy migration-mediated surface quenching, resulting in the alleviation of surface quenching at the nanoscale through heating. Since the thermal expansion coefficient of nanocrystals is usually size-dependent, the abnormal thermal enhancement of UCL originating from the suppression of energy-migration-induced surface quenching is correspondingly size-dependent [25,30,58,59].

It is noteworthy that the sensitizer and activator are randomly distributed within UCNP. As lattice expansion increases the separation between sensitizer ions (Yb^3+^), it simultaneously widens the distance between the sensitizers (Yb^3+^) and activator ions (Er^3+^, Eu^3+^, Tm^3+^, Ho^3+^, etc.). Since the energy transfer rate between the sensitizer and activator ions is inversely proportional the sixth power of their separation [8,9,10], it is anticipated that the energy transfer rate from Yb^3+^ to activators will decrease with increasing temperature due to this enlarged separation. This is evidenced by the shortened lifetime of UCL coupled with the prolonged lifetime and enhanced intensity of Yb^3+^ DSL emissions with increasing temperatures [25,44,50,51,56,58]. Given that the thermal expansion coefficient of nanocrystals is size-dependent and more significant for smaller particles [58,116], it is expected that the degree of decrease in the energy transfer rate from the sensitizer to activator increases with decreasing particle size.

**Figure 10:**
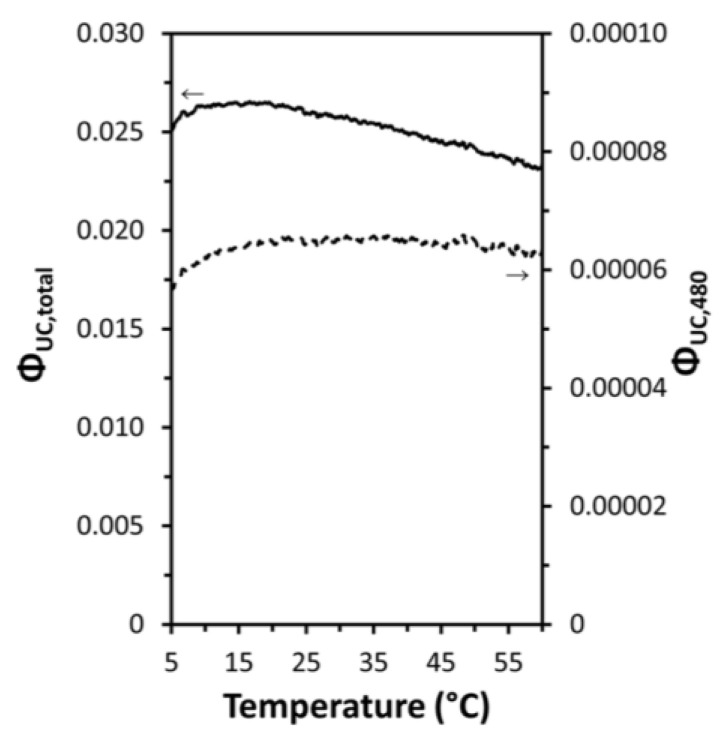
Temperature dependence of the total upconversion QY (solid line, left axis) and of the upconversion QY of the blue 480 nm band (dashed line, right axis) of LiYF_4_:Yb^3+^,Tm^3+^ UCNP in toluene recorded in Leiden; λ_exc_ = 969 nm, P_exc_ = 5.0 W·cm^−2^, [UCNP] = 5 mg·mL^−1^. Reproduced with permission from Ref. [17]. Copyright (2018) The Owner Societies.

As previously mentioned, DSL of Yb^3+^ and UCL of Ln^3+^ (Ho^3+^, Er^3+^, Tm^3+^) are competing processes that jointly depopulate the intermediate excited state of Yb^3+^ (^2^F_5/2_). The QY of UCNP is governed by a subtle balance between the impact of these different relaxation and energy-transfer processes. Even when the surface quenching is suppressed by lattice thermal expansion, it is unlikely that the QY of UCNP will increase at elevated temperatures due to the size-dependent increase in inter-ion separations between the sensitizer and the activators. If suppression of the surface-related quenching results in an enhanced energy transfer from Yb^3+^ to Ln^3+^, like the case of coating an inert shell on core UCNP, an increase in UCL lifetime with increasing temperature is expected [5,10,11,12,13]. This can also be demonstrated by the temperature dependence of the total upconversion QY and of the upconverted QY of the blue 480 nm band of LiYF_4_:Yb^3+^, Tm^3+^ UCNP measured using absolute integrating sphere setups in three European research groups, as depicted in Figure 10 [17].

(iii) The suppression of the OH vibration-induced de-excitation of the Yb^3+ 2^F_5/2_ state due to H_2_O desorption at high temperatures [44,50,51,55,56,57].

The rationale behind this mechanism is as follows: Nanoparticles are characterized by a large surface-to-volume ratio, and most quenching processes occur on the nanoparticle’s surface. In particular, surface-adsorbed water molecules induce significant quenching of upconversion emission by introducing high-energy oscillation modes, such as O-H vibration [10]. An increase in temperature typically leads to the desorption of these water molecules, thereby alleviating surface quenching. This, in turn, counteracts temperature quenching and may ultimately enhance UCL at higher temperatures [44]. The adsorption and desorption of moisture on the nanoparticle’s surface under ambient conditions have been confirmed by TG and FT-IR techniques [28,31,61].

**Figure 11:**
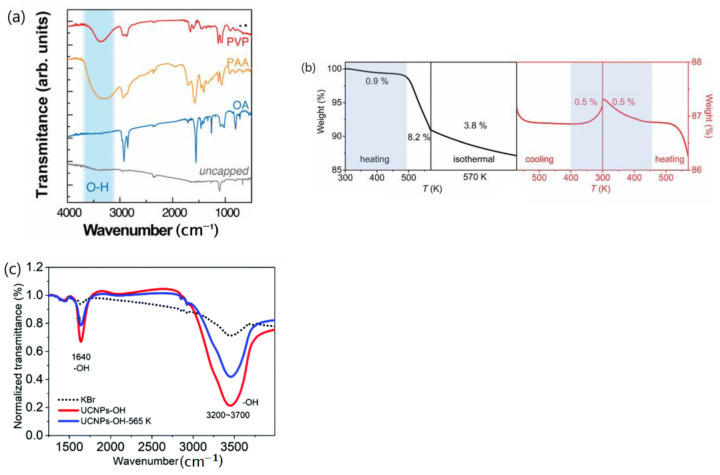
(**a**) FTIR transmittance spectra of the uncapped UCNPs and UCNPs with different capping ligands. Reproduced with permission from Ref. [57]. Copyright (2021) The Royal Society of Chemistry. (**b**) Thermogravimetric analysis of NCs in four stages: heating from 300 to 570 K at the rate of 5 K min^−1^ of a fresh NC sample; isothermal measurement at 570 K for 10 min; cooling from 570 to 300 K at the rate of 20 K min^−1^, and heating up again. The blue areas represent the adsorption or desorption of water on the NC surface. Reproduced from Ref. [28] under terms of the CC BY 3.0 license. Copyright (2019) the Owner Societies. (**c**) Room temperature FTIR spectrum of the OH coated UCNP before and after temperature-dependent measurement at 565 K. Reproduced with permission from Ref. [24]. Copyright (2019) The Royal Society of Chemistry.

Despite been adopted by multiple research groups [24,55,56,57], this mechanism has sparked some controversy [58,59,71]. Firstly, the thermal mitigation of surface quenching by H_2_O desorption fails to explain the thermal enhancement of UCL observed at cryogenic temperatures [26], as water desorption at atmospheric pressure is unlikely to occur at such low temperatures. Secondly, this mechanism also struggles to account for the thermal enhancement UCL observed in UCNP featuring an active-core/inert-shell architecture [59,71], as well as in UCNP capped with hydrophobic oleic acid (OA) [57]. In the case of the active-core/inert-shell UCNP, the inert shell effectively inhibits surface quenching. For the OA-capped UCNP, minimal water adsorption is evident from FTIR analysis, as illustrated in Figure 11a. Thirdly, this mechanism cannot explain the core-size dependence of thermal enhancement UCL in the inert-core/active-shell UCNP [58]. Moreover, this mechanism can hardly explain the excitation power-density-dependent thermal enhancement observed by Shao’s group [44]. Fourthly, it fails to elucidate the good reversibility of emission intensity enhancement during heating and cooling cycles, as shown in Figure 5a,b.

Given the sample size involved in temperature-dependent spectral measurements relative to the particle size, if UCL enhancement arises from thermal mitigation of surface quenching through H_2_O desorption, it should not be reversible. Although the adsorption and desorption of moisture on the nanoparticle’s surface are generally reversible and have been confirmed using TGA experiments, the following reasons explain why reversibility is not expected in this context:

(1) At a specified elevated temperature, such as 90 °C, the moisture content in the UCNP during heating is expected to be greater than that during cooling. This is because water desorbed from the phosphor’s surface at the temperatures above 90 °C cannot be re-adsorbed reversibly at this temperature; re-adsorption typically occurs at lower temperatures. A TGA experiment by Meijerink’s group on Yb^3+^/Ln^3+^ (Ln = Tm, Ho, Er) co-doped NaY(WO_4_)_2_ UCNP during the heating, cooling, and reheating cycles in air within a temperature range of 300–570 K indicated that, during cooling from 570 to 300 K, there was a significant weight increase due to water adsorption below 370 K. However, during reheating, continuous weight loss due to moisture desorption occurred even above 400 K [28], as demonstrated in Figure 11b.

(2) The UCNP powder samples used in the temperature-variable emission spectral measurement is generally around 1–2 mm in depth [48,51,82,119]. Considering the variation in excitation light penetration depth based on the activator doping concentration, for the UCNP powder with a particle size of, for example, 50 nm, at least 10 layers of the sample may be involved in the measurement and contribute to the emission spectral intensity recorded. During the cooling in heating and cooling cycles, moisture re-adsorption on the top surface layer of UCNP may easily occur. However, achieving equilibrium in moisture re-adsorption on deeper layers of densely packed powders during cooling is challenging. This can be demonstrated by the room temperature FTIR spectrum of the OH-coated NaGdF_4_:20%Yb^3+^,0.5%Tm^3+^ UCNP before and after temperature-dependent spectral measurement reported by Qiu et al., as displayed in Figure 11c [24]. It can be seen in Figure 11c that intensity bands at OH absorption bands between 1640 cm^−1^ and 3200–3700 cm^−1^ are notably reduced after temperature-dependent spectral measurement.

Given these two reasons, it seems reasonable to deduce that the moisture content in UCNP at any temperature during the heating and cooling cycles of the sample under ambient atmosphere is unlikely to be the same. Consequently, if the enhancement of UCL is caused by the thermal mitigation of surface-quenching by H_2_O desorption, the magnitude of UCL enhancement in the heating and cooling branches and across different thermal cycles should not be well reversible.

(iv) Surface-phonon-enhanced energy transfer [27,60,61,62].

This mechanism was proposed based on experimental observations that revealed a significant thermal enhancement of Tm^3+^ UCL in Yb^3+^/Tm^3+^ co-doped β-NaYF_4_ UCNP with high Yb^3+^ concentrations and core-only structures. Conversely, the thermal quenching of Tm^3+^ UCL was detected in the samples featuring core/shell architectures, those annealed at high temperatures, or those in the form of micro-rods. Fourier Transform Infrared (FTIR) and Raman spectroscopic measurements confirmed the presence of a long chain of oleic acids (OA) on the surface of core-only UCNP. The authors suggested that an efficient energy transfer process occurred with the involvement of “surface phonons” generated by the oxygen moiety-chelating Yb^3+^ ions, [Yb···O]. An increase in temperature led to the creation of more “surface phonons,” enhancing UCL brightness. By reducing the size of the nanoparticles to increase the surface-to-volume ratio, more [Yb···O] coordination sites participated in upconversion, resulting in amplified enhancement at higher temperatures [27]. Liu and Liang elaborated that the essence of the “surface-phonon”-assisted enhancement mechanism was the broadening of the Yb^3+ 2^F_5/2_ state at high temperatures, which reduced the energy mismatch between the 4f-4f transitions of Yb^3+^ and activators [62]. However, this mechanism has sparked debate among several authors [28,30,44,58].

(1) Wang and co-workers raised concerns about the existence of a critical temperature threshold beyond which efficient [Yb···O]-Ln^3+^ pairs start to form, since the surface vibration could only result in the optically inactive Yb^3+^ and Ln^3+^ ions within the dark layer at room temperature according to the experimental results [30]. Furthermore, Wang and colleagues stressed that the surface-phonon-assisted energy-transfer mechanism failed to explain the reported increase in intensity accompanied with the prolonged lifetime of Yb^3+^ DSL with temperature [25,44,50,51,56,58,79]. Since the DSL (Yb^3+^) and UCL (Ln^3+^) processes directly compete with the nonradiative transition of Yb^3+^ ions, an increase in energy transfer from Yb^3+^ to activator Ln^3+^ at higher temperatures should decrease the intensity and decay time of Yb^3+^ DSL [30].
(2) Meijerink and co-workers pointed out that surface-phonon-assisted energy-transfer mechanism could not explain the luminescence thermal quenching of OA ligand-stabilized Yb^3+^/Ln^3+^ co-doped UCNP when the temperature variable spectral measurement was conducted in dry Ar and N_2_ atmosphere [28,44]. It also failed to explain the thermal quenching of UCL observed in the bulk NaY(WO_4_)_2_:Yb^3+^/Ln^3+^ (Er^3+^, Ho^3+^, Tm^3+^) samples [28]. Since the Yb–O bond was embedded in the oxidic host like NaY(WO_4_)_2_ [28,44].
(3) In addition, multiple research groups have reported temperature-variable spectral measurements using colloidal suspensions of UCNP, showing luminescence thermal quenching of OA ligand-stabilized Yb^3+^/Ln^3+^ co-doped UCNP [51,81,112]. The surface-phonon-assisted energy-transfer mechanism encounters difficulties in explaining this phenomenon, as the interaction between Yb^3+^ and surface OA ligands was maintained in organic solvent during the spectral measurements.
(4) The surface-phonon-assisted energy-transfer mechanism could hardly give a reasonable explanation for the observed thermal enhancement of UCL in ligand-free and inert-shell coated UCNP, as exemplified in Figure 2 [22,47,60,71,118]. This is attributed to the absence or inhibition of the interactions between Yb and surface ligands due to their protective inert shells.

(v) Thermal-induced trapped electron release [22,57,63,64].

The proponents of this mechanism argue that the notion of defect-assisted Ln^3+^ UCL emission is rooted in the principles of long-persistent phosphors, which harness energy through specially crafted defect states and subsequently re-emit photons facilitated by thermal activation. The creation of defects via the aliovalent substitution of host lattice with Ln^3+^ has been corroborated through XPS characterization and density functional theory (DFT) calculations [22,63]. The authors proposed that, at room temperature, a portion of the absorbed energy is captured by the nearby defect state. As the temperature rises, akin to long-persistent luminescence, the electrons in the defect state are thermally activated, transitioning to adjacent excited energy levels of activators via thermal activation and engaging in the UCL process. This results in an increase in the excited state electron population and, consequently, an enhancement in UCL emission [22].

It is crucial to emphasize that the mere presence of defects in UCNP does not automatically imply that these defects can store excitation light at low temperatures and transfer the absorbed light to UC-emitting Ln^3+^ ions upon thermal activation. These processes occur only when the energy levels of the defects align with both the energy of the excitation light and the energy levels of the Ln^3+^ ions. Notably, the energies absorbed by long-persistent phosphors typically consist of UV or short-wavelength visible light, rather than the NIR light used in UCL spectral measurement.

Furthermore, if UCL originates from thermally induced trapped electron release, it is expected that lifetime of UCL would be prolonged due to the time electrons spend in the trapped state before returning to the luminescence centers. Since the lifetime of UCL typically involves contributions from the lifetimes of the intermediate states, e.g., the sensitizer’s excited state lifetime and the energy migration between the different sensitizer ions [8,9]. Defects inducing the negative thermal quenching of the DSL of various phosphors have been extensively reported in the literature. In a prior publication, the author of this paper delved into the matter concerning Eu^2+^-doped phosphors and suggested that the purported negative thermal quenching (NTQ) of Eu^2+^-doped phosphors might be a misinterpretation [87]. There is zero direct evidence in the published literature [22,63] to support the assertion that thermally induced trapped electron release can lead to a thermal enhancement of UCL in terms of either absorption or lifetime.

## 5. Discussion

Based on the preceding discussion, it is prudent to observe that, in terms of the replicability and predictability of experimental results, drawing a definitive conclusion with confidence that thermal enhancement of UCL is an intrinsic property of fluoride UCNP remains challenging. Furthermore, few of the mechanisms proposed in the existing literature have been satisfactorily substantiated. Despite considerable efforts devoted to this issue, a coherent and persuasive perspective is still lacking to adequately elucidate the intricate experimental observations associated with the thermal enhancement (or NTQ) of UCL in fluoride UCNP.

In my opinion, the fundamental reason for this dilemma is that all of these explanations were based on the assumption that variations in UCL spectral intensity with temperature are synonymous with variations in the QY of the given UCNP, influenced solely by the single nanoscale surface-related mechanism they discussed. This premise is flawed, as it overlooks numerous extraneous factors that could potentially contribute to variations in UCL spectral intensity across different temperatures. These include alterations in excitation power density stemming from the absorption of excitation light by adsorbed water and the desorption of water at higher temperatures, as well as shifts in measurement setup geometry due to lattice thermal expansion, exacerbated by laser-induced local heating and a steep increase in practical sample temperature, among others. By focusing solely on one aspect, the bigger picture is overlooked, which explains why none of the proposed mechanisms have been conclusively substantiated.

### 5.1. Factors Contributing to Changes in UCL Intensity with Temperature

Below is a brief discussion regarding the factors that may impact the temperature-dependent UCL spectral intensity. Additionally, it addresses the concurrent phenomena occurring during spectral measurements across varying temperatures, which may influence UCL intensity, but have thus been overlooked in the aforementioned literature.

It is well-known that photoluminescence can occur only when a phosphor absorbs light. Hence, the emitted intensity, denoted as *I_e_*_m_, is proportional to the absorbed intensity and QY (*η*) of the phosphor in question. This relationship can be mathematically expressed in terms of the intensity of the incident light (*I*_0_) and the intensity of the light transmitted through the sample (*I*_T_) as follows [120]:

*I*_em_ = *η* (*I*_0_ − *I*_T_) (1)

where the intensities *I*_0_ and *I*_T_ are given in photons per second, and *η* is the QY defined as the ratio between the emitted and absorbed photons.

In a luminescence experiment, only a fraction of the total emitted light is measured. The size of this fraction is influenced by many different factors, such as the numerical apertures for excitation and the solid angle for detection, the emission anisotropy, the scattering of the sample, and the sample geometry [89]. Consequently, the measured emitted intensity, denoted as *I*, can be expressed in terms of the incident intensity *I*_0_ as follows [19,120]:

*I* = *k*_g_ × *η* × *I*_0_ (1 − 10^−^*^εbc^*) (2)

where *k*_g_ is a geometric factor, *η* is the QY, *ε* is the absorptivity or molar attenuation coefficient of the phosphor at the excitation wavelength (M^−1^ cm^−1^), *b* is the path length of the light through the sample (cm), and *c* is the concentration of the activator in mol·L^−1^ or M. Equation (2) assumes that absorbance of the excitation light by any species other than the phosphor is negligible and that there is no self-absorbance of emitted light.

For DSL, the emission of a single photon can be initiated by the absorption of one high-energy photon. In the light conversion process, there are no nonlinear processes involved. The quantum yield (*η*) remains unaffected by the excitation intensity. The measured intensity (*I*) of the emission spectrum increases linearly with the increase in the incident intensity (*I*_0_). Conversely, for UCL emission which is excited by a multiphoton process, the quantum yield (*η*) is dependent on the incident intensity (*I*_0_). As a result, the measured intensity (*I*) of the emission spectrum typically increases at a rate that is greater than the rate of increase in the incident intensity (*I*_0_) [19,119,121,122].

It is evident from Equation (2) that the measured spectral intensity (*I*) of a UCNP is influenced not only by its QY (*η*) and absorptivity, but also by the geometric factor of the spectrofluorometer (*k*_g_) and the incident intensity of excitation light. In essence, apart from the changes in QY and absorptivity with temperature, which are intrinsic properties of a UCNP, variations in the geometric factor of the spectrometer and the incident intensity of the excitation light with temperature can equally lead to changes in the measured spectral intensity (*I*) of the UCNP at elevated temperatures. The inherent nonlinearity of UCL further amplifies the impact of these extrinsic factors on the spectral intensity compared to the downshifting luminescence. Therefore, to fully understand the reliability of experimental observations regarding the spectral intensities at varying temperatures, one should consider not only the change in QY of the UCNP with temperature, but also the concurrent phenomena that may arise during temperature-variable spectral measurements and potentially affect the UCL intensity. Below is a brief discussion on these factors and their influences on the UCL intensity.

1. Absorption of excitation light by the adsorbed water and its influence on the excitation power density at different temperatures

**Figure 12:**
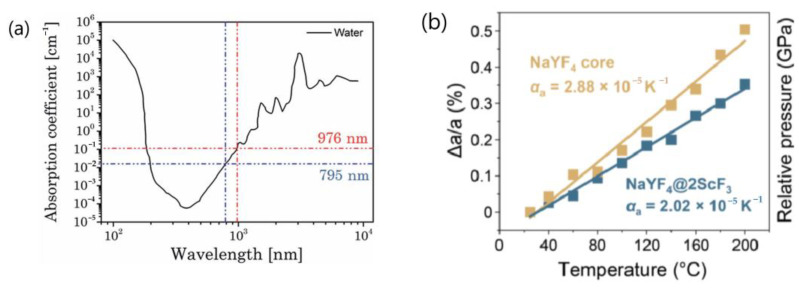
(**a**) Absorption coefficient of water in a spectral range of 100–10,000 nm. Values for the typical excitation of Yb^3+^ ions at 976 nm and Nd^3+^ ions are highlighted. Reproduced from Ref. [112] under terms of the CC BY 3.0 license. Copyright (2015) The Royal Society of Chemistry. (**b**) Lattice thermal expansion of the NaYF_4_ core and NaYF_4_@2ScF_3_. Reproduced from Ref. [116] with permission. Copyright (2024) American Chemical Society.

Multiple research groups have reported that water molecules exhibit strong absorption in both the UV and IR spectral regions [18,19,50,67,112], with a representative illustration provided in Figure 12a. Notably, the wavelength of excitation light employed in temperature-dependent spectral measurements for Yb^3+^-sensitized UCNP, approximately 980 nm, overlaps with the absorption band of water. This overlap suggests that water can competitively absorb the excitation light with Yb^3+^ during measurement. Consequently, the adsorption of water onto UCNP at low temperatures can diminish the power density of the excitation light incident to the sensitizer (Yb^3+^) and/or activator (Er^3+^), ultimately leading to a decrease in the QY and emission intensity of the UCNP, as outlined in Equation (2).

As the temperature increases, the adsorbed moisture progressively desorbs, resulting in a corresponding increase in the power density of the excitation light incident to the Ln^3+^ ion(s). This increase subsequently boosts both the QY and emission intensity of the UCNP. It is worth noting that the change in excitation power density have a more significant impact on the UCL intensity compared to the DSL intensity due to the nonlinear nature of the UCL process, in contrast to the linear DSL process [19,119,121,122]. In alignment with these observations, Shao and colleagues reported that the enhancement factors for DSL in Yb/Ln co-doped small nanoparticles at 423 K were lower than the corresponding UCL enhancement factors for the same phosphor [44,50]. This further substantiates the pivotal role of changes in excitation power density in determining the spectral intensity of UCNP.

2. Laser-induced local heating and temperature rise during measurement and associated issues

Due to the extremely low QY of UCNP, typically hovering around 1%, the majority of the absorbed excitation energy is either emitted as DSL from Yb^3+^ or Er^3+^ ions or dissipated as heat within the phosphor matrix. Consequently, during laser irradiation during measurements, the sample temperature rises. Numerous research groups have reported laser-induced local heating stemming from non-radiative relaxation accompanying the NIR laser energy upconversion [17,18,48,71,117,119,123,124,125,126,127]. For instance, Rettori et al. reported that the temperature of the small-sized NaGd_0.78_Yb_0.20_Er_0.02_F_4_@NaYF_4_ UCNP increased by 11 K before turning on the electrical power supply due to the heating produced by the incident laser irradiation [71]. Bednarkiewicz et al. reported irreversible spatially defined IR laser-induced annealing in a thin layer of dried colloidal solution of ultra-small (~8 nm) NaYF_4_:20%Yb^3+^/2%Er^3+^ nanocrystals, achieved under a localized, tightly focused beam excitation from a continuous wave 976 nm medium-power laser diode [124].

It is expected that the magnitude of the laser-indued temperature rise is influenced by both the doping concentration of Yb^3+^ ions and the QY of the phosphor. Specifically, higher Yb^3+^ doping and the lower QY of UCNP intensify the temperature rise due to enhanced absorption rates and increased non-radiative relaxation probabilities, respectively [122]. In addition, the power density of the excitation light also plays a role in determining the temperature rise [123,125]. The dependence of the emission intensity (or the QY) on the excitation power density is, in my opinion, intrinsically coupled to a change in the local temperature [71,126]. Furthermore, considering that the desorption of water is an endothermic process, the magnitude of temperature rise arising from laser-induced heating is likely influenced by the amount of adsorbed water. At low temperatures, the temperature rise in UCNP is expected to be smaller compared to higher temperatures due to a decrease in both the amount of adsorbed water and the QY of the UCNP at higher temperatures.

When subjected to the same power density, the temperature rise resulting from laser-induced local heating in UCNP is expected to surpass that of their bulk counterparts by a significant margin. This disparity is ascribable to the UCNP’s markedly lower QY, inferior thermal conductivity, and reduced specific heat capacity, as substantiated by experimental data [71]. Consequently, during the temperature-dependent UCL spectral measurement, the actual temperatures of UCNP and bulk material will deviate from the nominal temperatures specified at each measurement point. More specifically, UCNP are expected to exhibit higher actual temperatures compared to their bulk equivalents at each respective measurement point. Additionally, the divergence between nominal and actual temperatures for UCNP is poised to intensify at elevated nominal temperatures, owing to both the desorption of adsorbed water and decrements in QY. These temperature differences between UCNP and bulk materials, as well as within UCNP at low and high temperatures, undermine the reliability of temperature-dependent emission spectral intensity measurements due to the mismatch between practical and nominal temperatures.

3. Lattice thermal expansion-induced changes in sample volume and measurement geometry

In the majority of cases, the phosphor undergoes thermal expansion as temperatures rise, as an increase in energy leads to an expansion of the equilibrium spacing between atomic bonds. The thermal expansion coefficient of a compound is unique and influenced by its composition and structural rigidity [128,129]. For a given material, its thermal expansion coefficient can be affected by particle size [58,116,130,131]. Huang et al. reported that thermal expansion coefficient (α_a_) of cubic phase α-NaYF_4_:18%Yb^3+^/2%Ho^3+^ with a diameter of 6 nm derived from the temperature-dependent XRD was 2.88 × 10^−5^ K^−1^, as depicted in Figure 12b, a value evidently larger than that of the bulk counterpart (2.7 × 10^−5^ K^−1^) [116]. Lattice thermal expansion not only alters the energetic structure, energy transfer efficiency, vibronic properties, and decay kinetics of the phosphor, but also affects the volume and surface state the phosphor and the geometrical configuration of the measurement setup, subsequently influencing both the intensity of excitation light and the emission spectral intensity recorded by the spectrofluorometer. This topic has been previously addressed in the publications of the author [86,87,88,132,133,134,135,136]. Considering that the thermal expansion coefficient of fluoride UCNP is significantly larger than their bulk counterpart [58,116,130,131], and that UCNP experience higher temperature rises due to laser-induced local heating compared to bulk materials, it is expected that UCNP will undergo a more significant change in sample volume at high temperatures. This results in a considerable increase in the excitation intensity and alterations in the surface state of the UCNP sample, as well as in the geometric configuration of the setup. Consequently, the recorded signals may deviate from the intrinsic properties of the phosphor at elevated temperatures.

**Figure 13:**
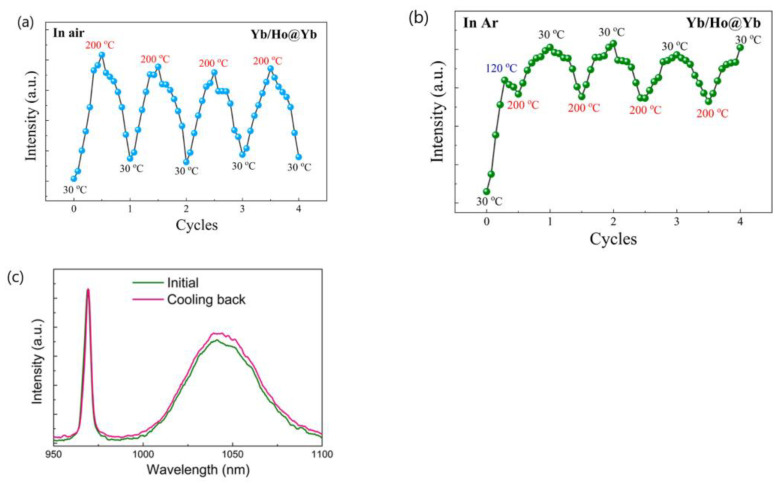
Integrated UCL intensity of NaGdF_4_:20%Yb/2%Ho@NaGdF_4_:20%Yb active-shell nanocrystals for four heating–cooling cycles in different atmospheres: (**a**) moist air, (**b**) dry Ar. Reproduced from Ref. [54] with permission. Copyright (2023) American Chemical Society. (**c**) Downconversion luminescence spectra of NaGdF_4_:Yb/Ho UCNPs before and after a heating–cooling cycle (λ_ex_ = 970 nm). Reproduced with permission from Ref. [51]. Copyright (2017) Royal Society of Chemistry.

The influence of measurement geometrical configuration on the spectral intensity is evident from the findings reported by Shao and colleagues [50,51,54]. Upon exposure to heating–cooling cycles either in air or in dry Ar atmospheres, a substantial increase in both the Ho^3+^ UCL intensity and Yb^3+^ DSL intensity was observed for NaGdF_4_:20%Yb^3+^/1%Ho^3+^ and NaGdF_4_:20%Yb^3+^/2%Ho^3+^@NaGdF_4_:20%Yb^3+^ UCNP at room temperature when compared to their pristine states, as illustrated in Figure 13 [50,51,54]. Given the minimal likelihood of enhancing the QY of either Ho^3+^ UCL or Yb^3+^ DSL in NaGdF_4_:20%Yb^3+^/1%Ho^3+^ UCNP subjected to heating and cooling cycles in ambient or dry Ar atmospheres during the spectral measurements, it is plausible that the observed increase in Ho^3+^ UCL and Yb^3+^ DSL intensities post-cycling is attributable to alterations in measurement conditions. Furthermore, considering the significant volume changes in the sample resulting from the combined effects of laser-induced size-dependent temperature elevations and lattice expansion at high temperatures, it is logical to deduce that the enhanced spectral intensity of these UCNP at elevated temperatures stems from similar factors rather than shifts in the QY of the phosphor. Specifically, the enhanced UCL spectral intensity of these fluoride UCNP at high temperatures may be linked to modifications in the sample’s surface state and/or adjustments to the spectroscopic geometrical configurations.

Based on the aforementioned discussion, it appears logically sound to conclude that, when utilizing a commercial spectrofluorometer to measure the temperature-dependent UCL spectral intensity of solid powders, the measurement conditions undergo alterations in tandem with temperature variations, despite the spectrofluorometer’s sample chamber and light source power remaining constant. These shifts in measurement conditions inevitably contribute to the fluctuations in spectral intensity with temperature [85,86]. The experimentally observed variation in UCL spectral intensity with temperature is likely a composite effect stemming from changes in both the QY of UCNP and the measurement conditions at elevated temperatures. Specifically, this includes the desorption of moisture, which results in an augmentation of excitation power density, and lattice thermal expansion exacerbated by significant temperature rises due to laser-induced local heating. These phenomena substantially increase the sample volume and modify the geometric arrangement of the setup. Considering that the rate of energy transfer from the sensitizer to the activator diminishes as the inter-ion separation increases, and that lattice thermal expansion leads to an augmentation of these separations, it seems improbable that the QY of the fluoride UCNP would increase at elevated temperatures.

Taking into account the aforementioned explanation, it becomes apparent why the thermal enhancement factor of UCNP increases as their particle size decreases. This is due to the size-dependency of various factors, including the adsorption amount of moisture, the temperature elevation stemming from laser-induced local heating, and the lattice thermal expansion coefficient. Analogously, the enhancement factor’s increase with higher doping concentrations of Yb^3+^ [22,27] and excitation power density [22,44] could be rationalized by the same reasoning. Higher doping concentrations of Yb^3+^ and excitation power densities result in a more substantial temperature rise due to laser-induced local heating, leading to a more pronounced change in the sample’s volume.

Understanding the distinct thermal enhancement behavior of active-core/inert-shell- and active-core/active-shell-structured UCNP is also straightforward. The latter generates more heat due to the enhanced absorption of excitation light facilitated by a higher concentration of sensitizers in the shell. Furthermore, considering that the thermal enhancement of UCL is primarily driven by changes in extrinsic measurement factors rather than the intrinsic properties of UCNP, it is unsurprising that the enhancement factor of a given UCNP reported by the same research group varies significantly. This variation is attributed to the influence of the UCNP sample’s packing state on the geometrical configuration’s temperature-dependent changes, which are difficult to maintain consistently across different measurements, even when conducted by the same analyst.

**Figure 14:**
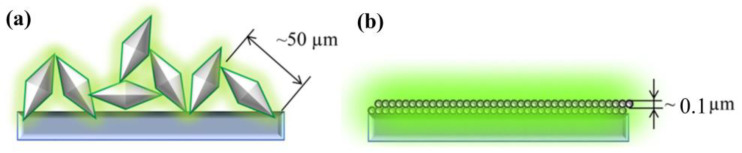
(**a**) Low packing density in a surface coated with microcrystalline phosphors. (**b**) Surface coated with a thin film of nanostructured phosphors. Reproduced from Ref. [137] with permission. Copyright (2015) American Chemical Society.

Using the same logical framework, the experimental observation that thermal quenching rather than the thermal enhancement of UCL was observed for the given UCNP when measured in an organic solvent dispersion [42,51,81,112] becomes readily comprehensible. On the one hand, the laser-induced temperature increase in the solution is substantially lower compared to that of a powder sample, owing to the reduced volume fraction of UCNP in the solvent and the solvent’s significant thermal capacity to dissipate heat from the UCNP [123]. On the other hand, the geometrical configuration of the setup for the UCNP solution contained within a quartz cuvette is anticipated to undergo minimal changes compared to the densely arranged powder sample. Similarly, the morphology-dependent thermal enhancement of UCL reported by Shao and co-workers, which demonstrated the thermal enhancement of UCL for small NaYF_4_:20%Yb^3+^/2%Ho^3+^ nanoparticles but thermal quenching for the nanorods [44], can be rationalized based on analogies. On the one hand, nanorods typically possess a lower packing density compared to nanoparticles, as illustrated in Figure 14 [137]. Consequently, the increase in the volume of loosely packed nanorod samples is expected to be smaller, potentially due to densification through the filling of interparticle empty spaces. On the other hand, the temperature rise due to the laser-induced local heating of nanorods is expected to be smaller than nanoparticles due to their relatively good thermal conductivity and high QY [71]. These combined factors account for the observed differences in the thermal behavior of UCL between the nanoparticles and nanorods.

QY is a key metric in lighting technology and for the quantification of luminescent processes, indicating how many photons are emitted with respect to the number of absorbed photons. The determination of QY using optical techniques can be approached via two methodologies: a relative method utilizing a fluorescence spectrometer to measure the spectral intensity of the sample relative to a reference standard with a known QY and absorption factors under identical measurement conditions, or an absolute method employing an integrating sphere to directly count the number of emitted photons per absorbed photon [17,18,19,80,85,89]. The main differences between the relative and absolute determination of fluorescence QY originate from the limitation of conventional fluorescence spectrometers, which can only capture a fraction of the emitted light. This fraction varies based on multiple factors, including emission anisotropy, sample scattering, and sample geometry, which are instrument-specific and thus difficult to quantify. Therefore, the relative method necessitates the use of a standard with a known QY and optical properties that closely resemble the sample under investigation. Given the fact that UCL arises from the sequential absorption of two or more photons, UCL intensity correlates nonlinearly with excitation radiant-power density [121,122]. Furthermore, the absence of a reliable standard with a known QY, power dependence, and matching absorption and scattering properties at the excitation wavelength in the NIR region complicates the use of a relative method for UCL. Consequently, it is advisable to adopt an absolute measurement approach to determine the QY of UCNP. Multiple independent research papers have emphatically underscored the necessity of determining the QY of UCNP with an absolute method under different excitation densities [17,18,80,85,89,119,138,139].

Investigating the luminescence thermal stability of UCNP by comparing the temperature-dependent UCL spectral intensity of given phosphor is essentially studying how the QY varies with temperature using a relative method. This method is predicated on the assumption that changes in the integrated emission spectra with temperature equates variations in the QY of the phosphor under investigation. However, as previously mentioned, the relative method is unreliable for determining the QY of UCNP, even under isothermal conditions. Variations in temperature can lead to changes in excitation power density, surface state, and/or geometrical configurations due to the desorption of moisture and thermal expansion of the UCNP, further compromising the reliability of the obtained results [86,87,88]. Considering the nonlinearity of the UCL process, a very small change in the excitation density or geometrical configurations can significantly impact the emission spectral intensity recorded, even if the QY of the UCNP maintains unchanged. Additionally, the lattice’s thermal-expansion-induced changes may exhibit varying degrees of reversibility depending on the packing state of the sample. This is because the volume of the packed particles for spectral measurement is composed of both the particle volume and the gaps between particles. While the volume change in separate particles due to lattice expansion is reversible, the changes in the gaps between particles may vary with different packing density, leading to different degrees of reversibility.

Quantitative measurements of luminescence spectra across various temperatures are conceptually simple tasks, and yet these procedures are subjected to several pitfalls that can lead to significant errors [85]. The electric signal produced using a spectrofluorometer is linked to the overall luminescence intensity through a number of instrumental factors, such as the intensity of the exciting source, instrumental optics, and signal amplification, which can frequently hide artifacts in the data. Furthermore, numerous inherent methodological issues are frequently overlooked, resulting in measurements that are unreliable and low-quality [86,87]. The size-dependent physicochemical properties of UCNP further complicate matters, as they impart a size dependency to the measurement error associated with emission spectral intensity, as detailed below:

(1) The moisture present on UCNP exhibits strong absorption in the NIR region, which can divert the power density of the excitation light incident to the sensitizer, thereby influencing the intensity of the UCL. Furthermore, the moisture on UCNP can retard the laser-induced temperature rise. Given that the quantity of moisture adsorbed at room temperature depends on the surface-to-volume ratio of the UCNP, the moisture-related influence on the temperature-dependent spectral intensity is inherently size-dependent.
(2) Laser-induced local heating is another factor influencing the reliability of spectral intensity. The magnitude of the laser-induced temperature rise is contingent upon the doping-concentration of Yb^3+^ ions and the QY of the UCNP under the constant excitation power density. Given that the QY of UCNP is dependent on particle size and decreases as particle size diminishes, it is anticipated that the impact of laser-induced temperature rise on the emission spectral intensity will also exhibit a size-dependent behavior.
(3) Changes in sample volume and surface state due to lattice thermal expansion can lead to alterations in the excitation power density and geometrical configuration of the measurement, ultimately influencing the spectral intensity recorded. The extent of these changes, while changes in sample packing density are not considered, depends on both the thermal expansion coefficient and the temperature increase. Given that both the thermal expansion coefficient and the laser-induced temperature rise exhibit size-dependence and tend to increase with decreasing particle size, it is expected that the influence of the lattice thermal expansion on the emission spectral intensity will also be size-dependent.

In light of the aforementioned reasons, comparing the emission spectral intensity at elevated temperatures to its counterpart at lower temperatures fails to accurately delineate the alteration in light conversion efficiency for specific UCNP. As a result, any mechanisms inferred from such uncertain findings would be challenging to validate. To forestall any potential misleading or impediments to genuinely insightful research into the mechanisms governing the thermal behavior of UCNP, it is imperative to target the utmost attention to the replicability and reliability of experimental observations when investigating temperature-dependent luminescence in these phosphors.

### 5.2. Factors Influencing Quantum Yield in Yb^3+^-Sensitized Upconversion Luminescence Phosphors and Challenges in Its Measurement

Quantum yield (QY), also referred to as “quantum efficiency” (QE), serves as a direct measure for assessing the intrinsic efficacy of a phosphor in light conversion processes. In Yb^3+^-sensitized UCL phosphors, the QY is dictated by the interplay between the energy transfer upconversion (ETU) rate and the linear decay rate that governs the depopulation of the intermediate state of the activator involved in the UCL process. Consequently, the QY exhibits a dependence on the excitation power density [121,122]. The energy transfer between the sensitizer and activator is predominantly regarded as a dipole–dipole interaction, where the energy transfer rate is proportionally to R^−6^, with R representing the distance separating the two ions. As a result, the spacing between the sensitizer and activator ions emerges as a critical factor influencing the QY of the UCL phosphor. At a constant temperature, the distance between two Ln^3+^ ions depends on their doping concentrations within the host. Furthermore, the distance between Ln^3+^ ions in a given phosphor may undergo variations due to temperature-induced expansion or contraction. These effects may induce change in energy transfer efficiency in specific circumstances. Notably, significant lattice expansion has been observed in fluoride UCNP at higher temperatures [116], making it unlikely that the energy transfer rate between the sensitizer and activator in fluoride UCNP would be enhanced at elevated temperatures. Although, theoretically, spectral broadening caused by vibronic coupling could potentially compensate for the energy mismatch between the donor and acceptor, this is not sufficient to overcome the distance-related decrease in energy transfer rate. Therefore, the observation of thermally enhanced UCL spectra is unlikely to be attributed solely to the enhanced light conversion efficiency of fluoride UCNP.

Measurements of UCL QY are inherently challenging, even under isothermal room temperature conditions, due to many factors such as the nonlinearity of the system, laser-induced local heating and its associated issues, etc. Employing a relative method with a spectrofluorometer to measure the emission intensity of the sample compared to a standard of known QY under the same conditions is unambiguously unreliable due to the scarcity of reliable standards with optical property and power dependence in the NIR region that closely match those of the investigated sample. Even minor changes in experimental parameters, such as fluctuations in laser power or wavelength, can induce substantial variations in the measured QY. Furthermore, temperature variations can result in changes in the excitation power density and the surface state and/or geometrical configurations due to moisture desorption and the lattice thermal expansion of the UCNP, further compromising the reliability of the obtained results.

In summary, the QY of Yb^3+^-sensitized UCL phosphors represents the intrinsic radiative efficiency of the system, which is influenced by multiple factors, including ion spacing and temperature-induced effects. Meanwhile, the measurement of UCL QY at different temperatures is fraught with challenges, necessitating the development of more robust and reliable measurement techniques to obtain accurate and consistent results.

## 6. Concluding Remarks

Over the last two decades, a vast number of articles have been published that report on the NTQ (or thermal enhancement) phenomena of UCNP with precisely controlled particle sizes and architectures, with numerous hypotheses proposing to explain the thermal enhancement phenomenon. However, it is crucial to acknowledge that these conclusions regarding thermal enhancement mainly stem from comparisons of UCL emission intensities at high and low temperatures without considering the variations in measurement conditions with temperature and their influence on emission spectral intensity. Essentially, this paper constitutes a study of QY variations with temperature using a relative method that assumes the unchanged absorption of excitation light by the sample and consistent measurement conditions as the temperature increases. This approach to studying the thermal quenching properties of phosphors by measuring emission spectral intensity at different temperatures may have historical origins. Researchers, therefore, seem to be unaware or unmindful of the fact that the relative method is unreliable for determining the QY of UCNP, even under isothermal conditions, due to system nonlinearity. Temperature variations also lead to changes in excitation power density, surface state, and/or geometrical configurations, further undermining the reliability of the results.

Consequently, it is not unexpected that inconsistent findings about thermal enhancement of a given UCNP have been reported, even within studies published by the same research groups. Few proposed mechanisms have been definitively validated as the true underlying cause of thermally enhanced UCL intensity. In our opinion, a critical barrier to obtaining comparable and replicable experimental result lies in the absence of standardized experimental protocols and measurement conditions, including the experimental setup, the excitation light’s wavelength and power density, the irradiation duration prior to spectral acquisition, the mathematical normalization of spectral intensities, etc. This lack of reliable, reproducible data poses a significant challenge to identifying the true mechanism, as fluorescence measurements inherently combine sample-specific characteristics with instrument-dependent variables—the latter of which are particularly sensitive to temperature-induced fluctuations. At elevated temperatures, concurrent phenomena—such as lattice thermal expansion, moisture desorption, and laser-induced local heating—can distort instrumental responses, affecting parameters like excitation light intensity, detector sensitivity, and optical alignment. These effects are further exacerbated by sample-specific factors, including particle size distribution, thermal expansion coefficients, and packing density, all of which introduce variability into the measured emission intensity.

A comprehensive understanding of the fundamental aspects of temperature-dependent UCL in nanomaterials should be grounded in reliable experimental observations. Merely relying on the temperature dependence of emission intensity as a measure is inadequate for validating the thermal enhancement behavior of UCNP. Without QY measurements across varying temperatures, observed increases in UCL emission intensity with temperature have little to no practical value for the design and fabrication of thermally stable fluoride UCNP. The quantitative assessment of UCL emission spectra across varying temperatures requires a careful and detailed consideration of the nonlinearity inherent in the UCL process, along with the maintenance of consistent measurement conditions across different temperatures.

While measuring emission spectral intensity under different temperatures using spectroscopic techniques is relatively straightforward, validating the reliability of the results is extremely important, but not easy. It is important to note that the replicability of experimental observations is a crucial pathway to attaining confidence in scientific knowledge, although not the only one. It is our hope that, when reporting on temperature-dependent luminescence phenomena, particular attention be given to the strict rigor and replicability of experimental observations. Measurement results should be expressed in terms of estimated value and its associated uncertainty. In the scientific literature, the comparability of measurement results is a fundamental requirement, since high-quality data are essential in obtaining an authentic understanding of the underlying mechanisms. The plethora of proposed mechanisms, frequently based on inaccurate experimental observations, paradoxically impedes any meaningful advancement in understanding the genuine thermal behavior of UCNP.

## Figures and Tables

**Table 1:** A collection of experimental data on the temperature-dependent UCL intensity of lanthanide-doped fluoride UCNP.

Phosphor	*I*_X_	*I*_max_(T)/*I*(T_0_)	∆*T* (K)	Δ*I*/Δ*T* (%·K^−1^)	Exc. Power (Density), Wavelength *	Size and Architecture **	Ref.
β-NaYF_4_:20%Yb^3+^/2%Er^3+^	*I*_integrated_	2	298–358	3.33	0.178 W·cm^−2^, 975 nm	24 nm, core-only	[21]
β-NaYF_4_:18%Yb^3+^/2%Er^3+^	*I_i_*_ntegrated_	2	10–150	1.4	290 W·cm^−2^, 980 nm	25 nm, core-only	[41]
α-NaYF_4_:18%Yb^3+^/2%Er^3+^	*I*_integrated_	<1	10–400		290 W·cm^−2^, 980 nm	6 nm, core-only	
NaYF_4_:10%Yb^3+^/0.72%Er^3+^	*I*_540nm_	~40%	10–120	3.33	700 mW, 980 nm (in organic solvent)	120 nm length, 50 nm width	[42]
β-NaYF_4_:20%Yb^3+^/2%Er^3+^	*I*_integrated_	3	303–423	2.5	1.6 W·cm^−2^, 980 nm	~7 nm, core-only	[44]
β-NaYF_4_:20%Yb^3+^/2%Er^3+^	*I*_integrated_	<1	283–353		200 mW, 980 nm (in cyclohexane)	24.1 nm, core-only	[81]
β-NaYF_4_:20%Yb^3+^/2%Er^3+^@NaYF_4_	*I_i_*_ntegrated_	<1	283–353		200 mW, 980 nm (in cyclohexane)	27.2 nm, 28.9 nm, 33.2 nm and 40.2 nm (core/shell)	
α-NaYF_4_:20%Yb^3+^/2%Er^3+^	*I*_545nm_	<1	303–483		2 W·cm^−2^, 980 nm	75 nm, core-only	[83]
α-NaYF_4_:10%Yb^3+^/1%Er^3+^	*I*_integrated_	<1	84–364		980 nm	40 nm, core-only	[109]
β-NaYF_4_:20%Yb^3+^/2%Er^3+^	*I*_integrated_	<1	303–523		20 W·cm^−2^, 980 nm	52 nm, core-only	[110]
β-NaYF_4_:40%Yb^3+^/2%Er^3+^	*I*_540nm_	<1	303–483		30 mW, 980 nm	29 nm, core-only	[111]
OA-capped β-NaYF_4_:Yb^3+^:Gd^3+^:Er^3+^	*I*_545nm_ I_660nm_	<1	288–328		130 mW, 976 nm (in cyclohexane)	30.2 nm,core-only	[112]
NaYF_4_:20%Yb^3+^/2%Er^3+^@NaYF_4_	*I*_integrated_	<1	160–300		0.1 W·cm^−2^, 980 nm	30 nm, core/inert shell	[113]
NaYF_4_:20%Yb^3+^/2%Er^3+^@ NaYF_4_	*I*_integrated_	<1	313–553		980 nm	23.5 nm, core/inert shell	[114]
β-NaGdF_4_:20%Yb^3+^/2%Er^3+^	*I*_integrated_	3.3	298–338	8.25	0.178 W·cm^−2^, 975 nm	7 nm, core-only	[21]
β-NaGdF_4_:20%Yb^3+^/0.5%Er^3+^	*I*_green_	11	303–393	12.22	1.0 W, 980 nm	10 nm, core-only	[25]
β-NaGdF_4_:20%Yb^3+^/2%Er^3+^	*I_i_*_ntegrated_	4	303–423	3.33	1.6 W·cm^−2^, 980 nm	19.3 nm, core-only	[49]
β-NaGdF_4_:39%Yb^3+^/1%Er^3+^	*I*_integrated_	2.1	303–423	1.75	5.0 W·cm^−2^, 976 nm	13.1 nm, core-only	[56]
NaGdF_4_:20%Yb^3+^/2%Er^3+^	*I*_integrated_	>1	483–573		980 nm	11–12 nm, core-only	[63]
		~2.2	293–413	1.83			
NaGdF_4_:20%Yb^3+^/2%Er^3+^@NaYF_4_	*I*_integrated_	3	300–400	3	1.3 W, 976 nm	9.0 ± 2.0 nm, core/inert shell	[71]
NaGdF_4_@NaGdF_4_:20%Ca^2+^/20%Yb^3+^/2%Er^3+^	*I*_integrated_	~10.9	293–413	9.08	980 nm	11 nm, core/shell	[115]
NaGdF_4_:20%Ca^2+^/20%Yb^3+^/2%Er^3+^	*I*_integrated_	~5.3	293–413	4.42	980 nm	14 nm, core-only	
NaGdF_4_@ NaGdF_4_:20%Yb^3+^/2%Er^3+^	*I*_integrated_	~3.44	293–413	2.87	980 nm	10 nm, core/shell	
NaGdF_4_:40%Yb^3+^@ NaGdF_4_:60%Yb^3+^/2%Er^3+^	*I*_integrated_	~8.24	293–413	6.87	980 nm	13 nm, core/shell	[46]
NaGdF_4_:20%Yb2%/Er@NaGdF_4_:20%Yb^3+^	*I*_green_	6.8	303–423	5.67	1.6 W·cm^−2^, 975 nm	14.3 nm, core/active shell	
NaGdF_4_:20%Yb^3+^/2%Er^3+^@NaGdF_4_	*I*_green_	<1	303–423		1.6 W·cm^−2^, 975 nm	14.9 nm, core/inert shell	[47]
NaGdF_4_:20%Yb^3+^/2%Er^3+^@NaGdF_4_	*I*_green_	7.7	303–423	6.42	1.6 W·cm^−2^, 975 nm	8 nm, core/inert shell	
NaGdF_4_:20%Yb^3+^/2%Er^3+^@NaGdF_4_	*I*_red_	5.1	303–423	4.25	1.6 W·cm^−2^, 975 nm	16.6 nm, core/inert shell	[47]
	*I*_integrated_	<1					
NaGdF_4_:20%Yb^3+^/2%Er^3+^@NaYbF_4_	*I*_green_	13	303–423	10.8	1.2 W·cm^−2^, 975 nm	~7.3 nm core/active shell	[52]
OA-capped NaYbF_4_:2%Er^3+^@NaLuF_4_:25%Yb^3+^	*I*_542nm_	4.74	298– 473	2.71	36 W·cm^−2^, 980 nm	~40 nm, core/inert shell	[59]
β-NaGdF_4_:20%Yb^3+^/2%Er^3+^@NaGdF_4_	*I*_integrated_	<1	303–423		1.6 W·cm^−2^, 980 nm	~14 nm, core/inert shell	[44]
NaGdF_4_@NaGdF_4_:20%Yb^3+^/2% Er^3+^	*I*_integrated_	7	303–423	5.83	1.1 W·cm^−2^, 980 nm	~12 nm (~9@3 nm) inert-core/active-shell	[58]
β-NaYF_4_:49%Yb^3+^/1%Tm^3+^	*I*_475nm_ *I*_450nm_	300 2000	300–453 300–453	196.1 1307.2	10 W·cm^−2^, 980 nm 10 W·cm^−2^, 980 nm	~10 nm, core-only	[27]
NaYF_4_:20%Yb^3+^/1%Tm^3+^	*I*_integrated_	2.9	298–398	2.9	975 nm	~22.1 nm, core-only	[48]
NaYF_4_:18%Yb^3+^/2%Tm^3+^	*I*_475nm_	16	300–453	10.45	0.5 W·cm^−2^, 980 nm	42 nm, core-only	[116]
	*I*_800nm_	<1	80–298		1.2 W·cm^−2^, 980 nm	6.1 nm, core-only	
NaGdF_4_:20%Yb^3+^/0.2%Tm^3+^	*I*_800nm_	9	303–423	7.5	1.0 W, 980 nm	10 nm, core-only	[25]
NaGdF_4_:20%Yb^3+^/0.5%Tm^3+^	*I*_475nm_	24.2	343–463	201.6	980 nm	11.6 ± 1.6 nm, core-only	[24]
β-NaGdF_4_:20%Yb^3+^/2%Tm^3+^	*I*_integrated_	4	303–423	3.33	1.6 W·cm^−2^, 980 nm	~7 nm, core-only	[44]
NaGdF_4_:20%Yb ^3+^/1%Tm^3+^	*I*_integrated_	6.2	298–398	6.2	975 nm	8.4 nm, core-only	[48]
NaGdF_4_:20%Yb ^3+^/1%Tm^3+^	*I*_475nm_	4.5	303–423	3.75	975 nm	~8.5 nm, core-only	[50]
β-NaGdF_4_:39%Yb^3+^/1%Tm^3+^	*I*_integrated_	17	303–453	11.33	5.0 W·cm^−2^, 976 nm	12.5 nm, core-only	[56]
NaGdF_4_:20%Yb^3+^/1%Tm^3+^	*I*_integrated_	10	303–423	8.33	3.4 W·cm^−2^, 980 nm	9.5 nm, core-only	[51]
	*I*_475nm_	25	298–423	20	4.7 W·cm^−2^, 980 nm		
NaGdF_4_:30%Yb^3+^/5%Tm^3+^@NaYF_4_	*I*_476nm_	8.3	300–415	7.22	1.3 W, 976 nm	6.6 ± 0.8 nm, core/inert shell	[71]
NaGdF_4_@NaGdF_4_:20%Yb^3+^/1%Tm^3+^	*I*_integral_	29	303–423	24.17	1.1 W·cm^−2^, 980 nm	~12 nm ~9@3 nm, inert-core/active-shell	[58]
NaGdF_4_:20%Yb^3+^/1%Tm^3+^	*I*_integrated_	11	303–423	9.17	1.1 W·cm^−2^, 980 nm	~12 nm, core-only	[27]
OA-capped β-NaYF_4_: 49%Yb^3+^/1%Ho^3+^	*I*_545nm_	7	303–403	7	0.5 W·cm^−2^, 980 nm	30 nm, core-only	
NaGdF_4_:20%Yb^3+^/2%Ho^3+^	*I*_integrated_	12.6	303–423	10.5	1.6 W·cm^−2^, 980 nm	~7 nm, core-only	[44]
NaYF_4_:2%Ho^3+^/20%Yb^3+^@NaYF_4_:40%Yb^3+^	*I*_540nm_	3	300–450	2.0	0.56 W·cm^−2^, 980 nm	42.7 ± 2.3 nm, core/active shell	[55]
NaGdF_4_:20%Yb^3+^/1%Ho^3+^	*I*_540nm_	52	298–423	41.6	4.7 W·cm^−2^, 970 nm	9.5 nm, core-only	[51]
NaGdF_4_:20%Yb^3+^/2%Ho^3+^	*I*_integrated_	52.1	298–398	52.1	975 nm	7.5 nm, core-only	[48]
β-NaGdF_4_:30%Ce^3+^/20%Yb^3+^/2%Ho^3+^	*I*_integrated_	7.5	298–398	7.5	975 nm	24.2 nm, core-only	[45]
	*I*_green_	10.2	303–423	8.5	1.6 W·cm^−2^, 975 nm	11.2 nm, core-only	
β-NaYF_4_:20%Yb^3+^/6%Nd^3+^	*I*_803nm_	136	298–433	100.74	0.13 W·cm^−2^, 980 nm	25 nm, core-only	[60]
β-NaYF_4_:20%Yb^3+^/6%Nd^3+^	*I*_803nm_	55	298–413	47.82	1 W·cm^−2^, 980 nm	20 nm, core-only	
β-NaYF_4_:20%Yb^3+^/6%Nd^3+^@NaYF_4_	*I*_803nm_	13	298–413	11.30	1 W·cm^−2^, 980 nm	30 nm, core/inert shell	
β-NaYF_4_:20%Yb^3+^/2%Nd^3+^	*I*_integrated_	1.8	297–420	1.46	10 W·cm^−2^, 980 nm	20 nm, core-only	[117]

*: Unless otherwise stated, the UCNP samples are in dry powder form. **: The average sizes of UCNP reported in the paper were predominantly determined using scanning electron microscopy (SEM) measurements without giving information regarding size dispersion.

## Data Availability

No new data were created.
